# The oxanorbornene approach to 3-hydroxy, 3,4-dihydroxy and 3,4,5-trihydroxy derivatives of 2-aminocyclohexanecarboxylic acid

**DOI:** 10.1186/1860-5397-2-9

**Published:** 2006-05-04

**Authors:** Ishmael B Masesane, Andrei S Batsanov, Judith A K Howard, Raju Mondal, Patrick G Steel

**Affiliations:** 1 Department of Chemistry, University of Durham, South Road, Durham, DH1 3LE, UK; 2 Department of Chemistry, University of Botswana, P/bag 00704, Gaborone, Botswana

## Abstract

The nitro oxanorbornene adduct derived from the Diels-Alder reaction of ethyl (*E*)-3-nitroacrylate and furan provides a versatile template for the stereoselective synthesis of hydroxylated derivatives of 2-aminocyclohexanecarboxylic acid (ACHC).

## Introduction

**Scheme 1:**
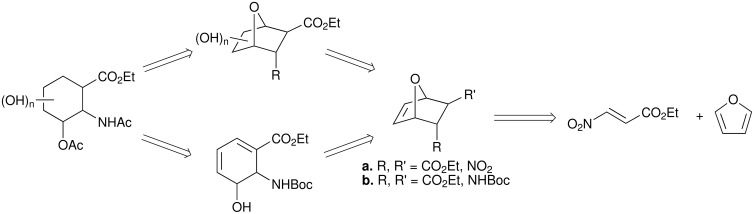
The furan-nitroacylate approach to poly hydroxylated ACHC derivatives

In recent years there has been a surge of interest in cyclic β-amino acids and this has been accompanied by the proliferation of procedures for their synthesis.[1] Much of the interest in these compounds emanate from the ability of oligomers or short polymers of cyclic β-amino acids to adopt well-defined secondary structures analogous to those of natural peptides. [2–9] Such oligomers therefore have a particular appeal for extending understanding of protein structure and stabilization. However, much of this work has focused on simple cyclic β-amino acids and analogous studies of substituted derivatives are less well developed. One reason for this is that simple stereocontrolled routes to such derivatives have not been defined. In this respect, we have been exploring the use of oxanorbornene adducts derived from the Diels-Alder reaction of ethyl (*E*)-3-nitroacrylate and furan as versatile intermediates in the synthesis of a range of novel hydroxylated derivatives of 2-aminocyclohexanecarboxylic acid (ACHC). In previous preliminary communications we have described the application of the *endo* nitro adduct to the synthesis of various polyhydroxylated analogues.[10–11] In this paper, we provide the full details of this work and additionally describe the stereoselective synthesis of further novel hydroxylated derivatives of ACHC using the *exo* nitro oxanorbornene adduct as the template. The basis of our approach to these poly hydroxylated cyclohexane β-amino acids was the recognition that the bicyclic oxanorbornene cycloadduct, derived from the Diels-Alder reaction between furan and ethyl nitroacrylate, represented a versatile template that could be elaborated to the desired targets via a variety of complementary oxidative processes coupled with a base promoted ring fragmentation, Scheme 1.

## Results and Discussion

### Oxanorbornene Synthesis

**Scheme 2:**
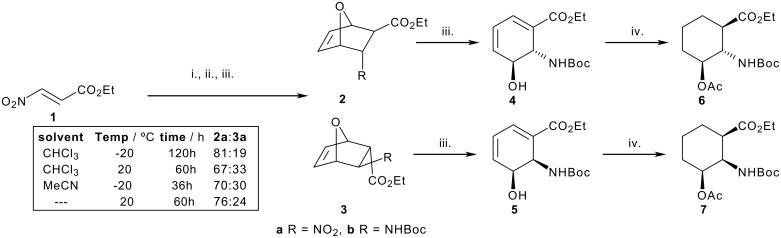
Reagents: i. Furan (see box); ii. Zn, HCl., EtOH then Boc_2_O, Et_3_N, 2b 89%, 3b 77%; iii. KHMDS, THF, -50°C, **4** 71%, **5** 69%; iv. Ac_2_O, Py then H_2_, Pd-C, **6** 75%, **7** 75%.

Efficient access to ethyl (*E*)-3-nitroacrylate **1** was achieved through a modification of the McMurry procedure involving the reaction of ethyl acrylate with N_2_O_4_ and I_2_ followed by careful elimination of HI with Hunig's base in ether.[12] With this sequence of reactions, **1** could be generated in 10–20 g batches, Scheme 1. Whilst this dienophile reacts with considerable regiocontrol (dominated by the nitro group) the stereoselectivity (endo:exo ratio) observed is frequently minimal. [13–15] Subjection of nitroacrylate **1** to cycloaddition reaction with furan in CHCl_3_ at room temperature gave a 2:1 mixture of cycloadducts favouring the *endo* nitro isomer **2a**, Scheme 2. Increased *endo* selectivity could be achieved by carrying out the reaction at -20°C for 5 days, giving a 4:1 mixture of the two isomers in 90% yield. Attempts to further enhance this ratio through the use of a range of Lewis acids or alternative solvents were not successful. Interestingly, Just and co-workers have reported the preference for the *exo*-nitro adduct when the reaction of methyl 3-nitroacrylate and furan was carried out without a solvent.[16] However, in our hands, this reaction afforded a 3:1 mixture in favour of the *endo*-nitro adduct.

The two isomeric cycloadducts were easily separated by column chromatography. Whilst initial attempts to reduce the nitro group were complicated by difficulties in the isolation of the resultant amine, an efficient one-pot conversion into the protected aminoesters **2b** and **3b** was achieved by reduction with Zn/HCl followed by addition of (Boc)_2_O and a large excess of ^i^Pr_2_NEt. At this stage, confirmation of the correct assignment of the relative stereochemistry for each isomer was realised by an X-ray crystallographic structure determination of the *endo* carbamate **2b** (see Supporting Information File 1 for details).

### 3-Hydroxy-2-aminocyclohexanecarboxylic acids

**Figure 1:**
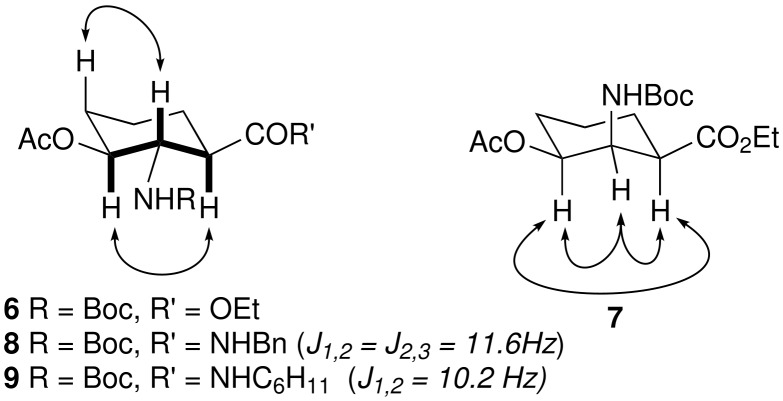
Selected NOESY Correlations for **6**–**9**

With the oxanorbornene template available in quantities we then explored the based promoted fragmentation needed to generate the 3-hydroxy-2-aminocyclohexanecarboxylate core. Related base promoted transformations have been described in the literature and our initial experiments followed these precedents. [17–20] Using carbamate **2b**, treatment with LDA or LHMDS appeared, by TLC, to give complete conversion extremely rapidly. However, on workup, considerable amounts of the starting ester were recovered. Similar observations have been reported in related systems.[21] Speculating that this was due to either a reversible process or a stabilised, highly coordinated enolate alternative procedures were then explored. Whilst attempts to enhance the fragmentation through the use of various rapid, mildly acidic, inverse quenches or various trapping agents (e.g. TMSCl, TMSOTf) were partially successful, albeit only on very small scale, the use of a less coordinating potassium counterion (KHMDS) allowed the isolation of the dihydroanthranilate esters **4** and **5** in 71% and 69% yield respectively together with variable amounts of ethyl 3-hydroxybenzoate. Simple reduction of the corresponding acetates with H_2_ over Pd-C proved to be highly diastereoselective affording the *anti-anti* and *syn-syn* 3-hydroxy-2-aminocyclohexanecarboxylate esters respectively. The stereochemistry was assigned by a combination of NMR techniques using J values supported by nOe correlations, Figure 1. For example, the amide derivatives of the *anti*-*anti* isomer **8** and **9** show distinctive large di-axial couplings for 1-*H*, 2- *H* and 3-*H*. Final confirmation of the stereochemistry was achieved by reduction and deprotection of a single enantiomer of carbamate **4**, obtained via resolution of bicyclic ester **2a**, to provide 2-amino-3-hydroxycyclohexane-1-carboxylate with identical spectroscopic and analytical data to that described in the literature ([α]_D_^21^ -32 (c = 1, H_2_O) lit: [α]_D_^21^ -35 (c = 1, H_2_O).[22] We attribute the stereochemistry of the reduction of **4** to the directing effect of the NBoc carbamate *vide infra*, whilst the reversal observed with **5** can be attributed to the severe steric crowding of the β-face by the three *syn* substituents.

### 3,4-Dihydroxy-2-aminocyclohexanecarboxylic acids

**Scheme 3:**
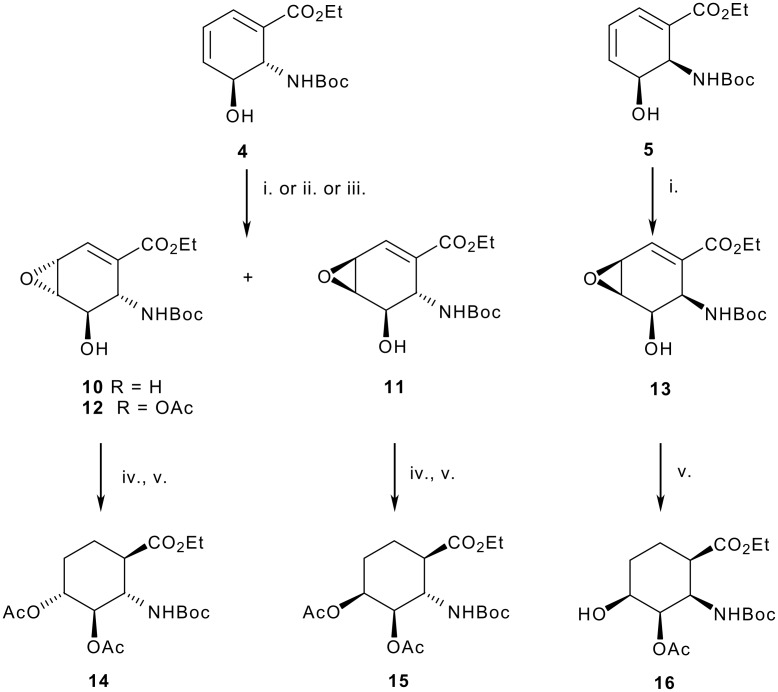
Reagents: i. mCPBA, CH_2_Cl_2_ **11** : **12** (9 : 1) 77%, **15** 82%; ii. Ac_2_O, Py then mCPBA, CH_2_Cl_2_ (**13** only) 65%; iii. mCPBA, MeCN, NaHCO_3_ **11** : **12** (1 : 2) 95%; iv. Ac_2_O, pyridine; v. H_2_, Pd/C, **14** 88% from **12**, **15** 84% from **11**, **16** 97%.

Having established an efficient entry to the oxanorbornenes and dienyl carbamates we then sort to exploit the alkene units to provide a convenient source of poly-hydroxylated cyclohexane β-aminoacids. Consequently, treatment of endo derived hydroxy diene with *m*CPBA in DCM afforded a separable 9:1 mixture of epoxides **10** and **11** favouring addition *syn* to the carbamate, Scheme 3. Highly selective epoxidations of cycloalkenyl amides have been described[23] and, consistent with a hypothesis that the mixture of **10** and **11** arise through competing H-bonding directing effects of the carbamate and hydroxyl groups, acetylation of the free hydroxyl group prior to oxidation enhanced the selectivity leading to exclusive formation of **12**. In a similar fashion, and in agreement with the idea of two directing groups working in concert, epoxidation of the *exo* derived diene **5** afforded exclusive formation of epoxide **13**. Speculating that disruption of any directing effects through the use of more polar solvents would lead to increased amounts of **12** a range of alternative solvents were screened including THF, DMF, MeOH, CF_3_CH_2_OH, MeCN. Of these, acetonitrile proving the most successful, albeit only providing a 2:1 mixture of isomers. Attempts to further enhance this reversal of selectivity using a variety of alternative procedures were not successful with most methods affording significant quantities of the corresponding protected anthranilic acid.

**Figure 2:**
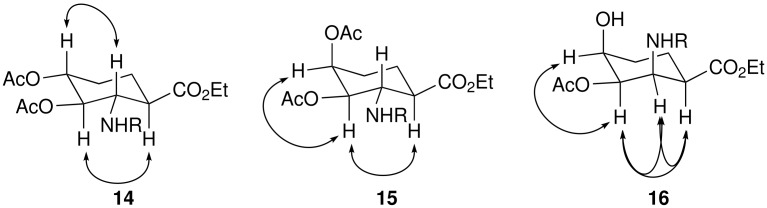
Selected NOESY Correlations for **14**–**16** (R = Boc)

Having introduced the epoxide function to either face of the diene unit we then sought conditions for regioselective cleavage. In this context, palladium mediated reactions of vinyl epoxides are known to proceed with allylic cleavage.[24] Subsequent addition of hydrogen to the intermediate π-allyl palladium complex would provide the desired 4-hydroxy-ACHC derivative. In agreement with this analysis, reaction of the epoxides with hydrogen in the presence of Pd-C afforded the corresponding 3,4-dihydroxy-2-aminocyclohexancarboxylate derivates **14–16** in excellent yield and diastereoselectivity. In each case the reduction afforded the product with a cis relationship between the 3-hydroxy group and C-1 carboxylate as ascertained by NOESY experiments, Figure 2. Whilst reactions of allylic electrophiles normally occur with *anti* attack by the Pd catalyst, the result using **12** suggests possible involvement of the carbamate. In this case the exception occurs with **13** which can be attributed to the severe steric crowding inhibiting such a co-ordination mode.

### 3,4,5-Trihydroxy-2-aminocyclohexanecarboxylic acids

**Scheme 4:**
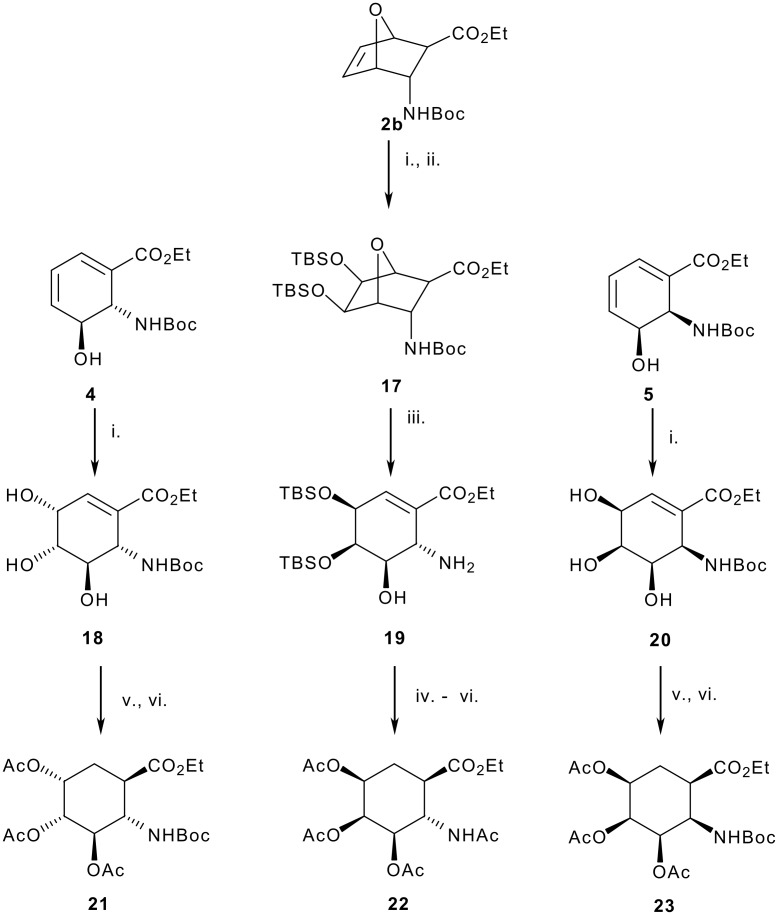
Reagents: i. OsO_4_, Me_3_NO.H_2_O, acetone **18** 78% from **4** ii. TBSCl, imidazole, CH_2_Cl_2_, **17** 66% from **2b**; iii. LiHMDS, THF, -50-25 °C, 48%; iv. ^n^Bu_4_NF, THF; v. Ac_2_O, pyridine; vi. H_2_, Pd-C, **21** 67% from **18**, **22** 51% from **19**, **23** 74% from **5**.

The final group of targets we wished to generate was the trihydroxy analogs. Initial attempts addressed the generation of the *syn* 4,5 set. Consequently, OsO_4_ mediated dihydroxylation and subsequent peracylation of dienes **4** and **5** proved to be *facio*-specific and afforded cyclohexenyl derivatives **18** and **20** in 75 % yield over the two steps, Scheme 4. Reduction, as previously, afforded the trihydroxy β-aminoacid derivatives in excellent yields.

At this stage it is instructive to address the interesting stereochemical outcome of this dihydroxylation reaction. Concurring with our results, Donohoe has reported that cyclic homoallylic carbamates give high levels of *syn* selectivity in the OsO_4_ mediated dihydroxylation reactions.[25–26] On the other hand, Kishi has established that the OsO_4_ mediated oxidation of cyclic allylic alcohols led preferentially to *syn*-*anti* triols.[27–28] On the basis of the results from our system where the two processes acted against each other, one can conclude that the homoallylic carbamate ability of stereocontrolling the OsO_4_ mediated reaction overrides that of the allylic hydroxyl group. Moreover, such directing effects are sufficiently strong that attempts using TMEDA/OsO_4_ combinations known to afford syn dihydroxylation of cyclic allylic alcohols were not successful in reversing the selectivity of the reaction with **4**.

In order to introduce the syn diol unit on the opposite face to the carbamate in **4** we returned to the oxanorborne cycloadduct in which electrophile additions are known to occur from the exo face. As predicted, smooth selective formation of the desired e*xo* diol **17** was achieved. Following double protection as a *bis* TBS ether, initial attempts to achieve the desired fragmentation using KHMDS failed. Fortunately, use of LiHMDS was successful albeit accompanied by the unexpected loss of Boc group. Similar strategies using either the free diol or the corresponding acetonide were not successful leading to extensive decomposition. Following TBAF promoted desilylation, peracylation and reduction afforded the trans β-amino acid derivative **22** as established by NMR experiments.

**Figure 3:**
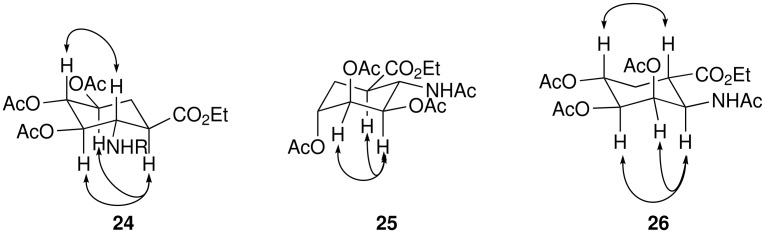
Selected NOESY correlations for **24** – **26**

Having prepared the 4,5-*cis* diol, we turned our attention to the synthesis of the corresponding *trans* diols. These could be prepared by simple hydrolytic cleavage of epoxides **10–13**. In line with this plan, treatment of **12** with aqueous perchloric acid led to a single *trans* diol, albeit accompanied by the loss of the Boc protecting group. Characterisation of the peracetylated derivative **27** suggested that nucleophilic attack had occurred at the allylic position. Support for this was confirmed by ring opening with ZnCl_2_ to afford the crystalline chlorohydrin **28** which was characterised by x-ray diffraction. Reduction of the double bond then afforded the expected all *anti* trihydroxy ACHC isomer **24** as confirmed by 1D and 2D NMR experiments. The observation of 10.0 Hz H_a_H_b_ coupling constants in the ^1^H NMR spectrum of **24** for the ring protons (H-1, H-2, H-3, H-4 and H-5) is consistent with *anti* diaxial relationships. Such assignments are supported by nOe interactions between H-1 and H-3, H-1 and H-5 and also H-2 and H-4, Figure 3.

**Scheme 5:**
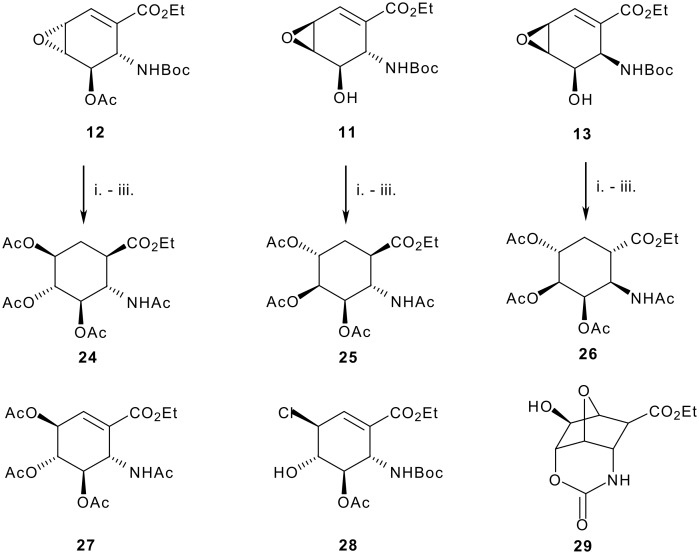
Reagents: i. HClO_4_, H_2_O/acetone; v. Ac_2_O, pyridine; iii. H_2_, Pd/C, **24** 71%, **25** 67%, **26** 71%

Similar treatment of epoxides **11** and **13** then afforded the corresponding *anti* 4,5 diols. Subsequent reduction of the double bond gave the *anti*-*syn*-*anti*-*anti* and *anti*-*syn*-*syn*-*anti* isomers **25** and **26** as the only detectable products, Scheme 5. In each case it is noteworthy that the reduction proceeded with delivery of hydrogen to the same face as that occupied by the carbamate and opposite to the 3-acetoxy group. As previously, the stereochemical relationships were initially assigned on the basis of 2D NMR experiments, particularly NOESY. Subsequently, the structure of **26** was confirmed by X-ray crystallographic analysis.

Attempt to generate the remaining epoxide isomer were confounded by an inability to overcome the directing influence of the carbamate and deliver the epoxide to the opposite face of diene **5**. Similarly, whilst the alternative regiochemistry in the epoxide ring opening product could be achieved exploiting neighbouring group participation by the NBoc group to afford the cyclic carbamate **29**, all attempts to induce fragmentation of this polycyclic compound have thus far proved unsuccessful.

## Conclusion

In conclusion, we have amplified the utility of the Diels-Alder adducts of ethyl (*E*)-nitroacrylate and furan by devising stereoselective routes to hydroxylated derivatives of 2-aminocyclohexanecarboxylic acid. The principal feature of this approach is the reliance on simple reactions exploiting substrate-controlled selectivity. In this respect, the ability of the carbamate group as a directing group is particularly noteworthy. The use of these hydroxylated cyclohexyl β-amino acids in the synthesis of short peptides is currently ongoing in our laboratory and will be described in due course.

## Supporting Information

File 1 Experimental procedures and spectroscopic data are provided for all new compounds including details of the X-ray diffraction studies for compounds **2b, 26** and **28**.
