# Supplementary material for: The oxanorbornene approach to 3-hydroxy, 3,4-dihydroxy and 3,4,5-trihydroxy derivatives of 2-aminocyclohexanecarboxylic acid
Source: Beilstein J Org Chem. 2006 May 4;2:9. doi: 10.1186/1860-5397-2-9 (PMC1524792; doi:10.1186/1860-5397-2-9)
Supplement: File 1 — Experimental procedures and spectroscopic data are provided for all new compounds including details of the X-ray diffraction studies for compounds 2b, 26 and 28. [file Beilstein_J_Org_Chem-02-09-s001.doc]

The oxanorbornene approach to 3-hydroxy, 3,4-dihydroxy and 3,4,5-trihydroxy derivatives of 2-aminocyclohexanecarboxylic acid

Ishmael B. Masesane,*^a,b^ Andrei S. Batsanov,^a^ Judith.A. K. Howard,^a^ Raju Modal^a^ and Patrick G. Steel^*a^

^a^Department of Chemistry, University of Durham, South Road, Durham, DH1 3LE, UK.

^b^Department of Chemistry, University of Botswana, P/bag 00704, Gaborone, Botswana.

Email: Ishmael B.Masesane- [masesane@mopipi.ub.bw](mailto:masesane@mopipi.ub.bw);

Patrick G. Steel- [p.g.steel@durham.ac.uk](mailto:p.g.steel@durham.ac.uk)

**ADDITIONAL MATERIALS**

**EXPERIMENTAL**

**Ethyl (*E*)-3-nitropropenoate 1**

N_2_O_4_ (9.5 ml, 271 mmol) was added to a stirred and cooled (0^o^C) mixture of ethyl acrylate (44.5 ml, 410 mmol) and iodine (31 g, 122 mmol) in diethyl ether (400 ml). The reaction mixture was stirred for 1 hour at 0^o^C and then at room temperature for 4 hours. The resulting dark solution was washed with saturated Na_2_S_2_O_3_ solution (5 x 200 ml). The aqueous layer was extracted with diethyl ether (200 ml) and the organic layers were combined and dried over magnesium sulphate. The solvent was removed under reduced pressure and excess ethyl acrylate was distilled off under *vacuo* at room temperature to give ethyl 2-iodo-3-nitropropanoate (31.1 g, 93 %) as a yellow oil. A solution of DIPEA (5.8 g, 45mmol ) in diethyl ether (10 ml) was then added dropwise to a vigorously stirred and cooled (0 ^o^C) solution of the yellow oil (10.0 g, 37 mmol) in diethyl ether (200 ml) and the mixture was stirred for 15 minutes. The resulting suspension was filtered through a plug of silica. The silica was flushed with 10 % diethyl ether in petroleum ether and the solvent was removed under reduced pressure to give an orange solid. The orange solid was passed through a short column of silica gel using petroleum ether/diethyl ether (9:1) as the eluent to afford title compound **1** (4.00 g, 73 %) as a yellow solid; Found: C, 41.35, H, 4.89, N, 9.66. C_5_H_7_NO_4_ requires C, 41.38; H, 4.86; N, 9.65; m.p 36-38 ^o^C; ν_max_ (KBr disk): 3415, 1736, 1638, 1542 cm^-1^; δ_H_ (200 MHz, CDCl_3_): 1.35 (3H, *t*, J = 7.0 Hz, OCH_2_CH_3_), 4.32 (2H, *q*, J = 7.0 Hz, OCH_2_CH_3_), 7.09 (1H, *d*, J = 13.6 Hz, H-2), 7.68 (1H, *d*, J = 13.6, H-3). δ_C_ (75 MHz, CDCl_3_): 13.9 (OCH_2_CH_3_), 62.4 (OCH_2_CH_3_), 127.6 (C-2), 148.9(C-3), 162.6 (C-1); MS (EI): *m/z* 145 (M^+^), 100 (100%).

**3-nitro-7-oxa-bicyclo[2,2,1]hepta-5-ene-2-carboxylate**:

Furan (5.4 ml, 74mmol) was added to a solution of α,β-unsaturated ester **1** (5.38g, 37mmol) in chloroform (20 ml) at 25^o^C. The reaction was stirred at 25^o^C for 16 h. Removal of the solvent under reduced pressure afforded a mixture of the *endo*-nitro isomer and the *exo*-nitro isomer (2:1) as a yellow oil. The oil was subjected to flash column chromatography using petroleum ether/diethyl ether (7:3) as the eluting solvent to afford the *Endo*-nitro adduct **2a** as a white solid (60%) and *Exo*-nitro isomer **3a** as yellow oil (30%). ***Ethyl endo-3-nitro-7-oxa-bicyclo[2,2,1]hepta-5-ene-exo-2-carboxylate* 2a** Found: C, 50.76; H, 5.14; N, 6.58. C_9_H_11_NO_5_ requires C, 50.70; H, 5.20; N, 6.57; mp 54-56^o^C; ν_max_ (KBr disk): 2982, 1722, 1587 cm^-1^; δ_H_ (300 MHz, CDCl_3_): 1.32 (3H, t, J = 7.2 Hz, OCH_2_CH_3_), 3.23 (1H, d, J = 3.0 Hz, H-2), 4.27 (2H, q, J = 7.2, OCH_2_CH_3_), 5.34 (1H, s, H-1), 5.48 (1H, d, J = 5.0 Hz, H-3), 5.54 (1H, dd, J = 5.0 and 4.0 Hz, H-4), 6.39 (1H, dd, J = 5.8 and 1.6 Hz, H-5), 6.73 (1H, dd, J = 5.8 and 1.6 Hz, H-6). δ_C_ (75 MHz, CDCl_3_): 14.4 (OCH_2_CH_3_), 49.2 (C-2), 62.4 (OCH_2_CH_3_), 79.3 (C-4), 83.5 (C-1), 84.5 (C-3), 133.9 and 139.1 (C-5 and C-6), 169.9 (CO_2_C_2_H_5_); m/z (CI) 231 (MNH_4_^+^, 100%). ***Ethyl exo-3-nitro-7-oxa-bicyclo[2,2,1]hepta-5-ene-endo-2-carboxylate* 3a** ν_max_ (Liq. Film): 2990, 1733, 1549 cm^-1^; δ_H_ (300 MHz, CDCl_3_): 1.28 (3H, t, J = 7.2 Hz, OCH_2_CH_3_), 3.94 (1H, t, J = 3.8 and 2.8 Hz, H-2), 4.16 (2H, J = 7.2 Hz, OCH_2_CH_3_), 4.82 (1H, d, J = 2.8 Hz, H-1), 5.32 (1H, d, J = 3.8 Hz, H-3), 5.50 (1H, bs, H-4), 6.54 (2H, m, H-5 and H-6). δ_C_ (75 MHz, CDCl_3_): 14.3 (OCH_2_CH_3_), 49.9 (C-2), 61.9 (OCH_2_CH_3_), 79.3 (C-4), 84.2 (C-1), 134.5 (C-6), 138.5 (C-5), 168.9 (CO_2_C_2_H_5_); m/z (CI) 231 (MNH_4_^+^); HRMS (CI): Found MNH_4_^+^, 231.1417. C_9_H_15_N_2_O_5_^+^ requires *M,* 231.1027.

**Ethyl *endo*-3-*tert*-butoxycarbonylamino-7-oxabicyclo[2,2,1]hept-5-ene-*exo*-2-carboxylate 2b**

Concentrated HCl (28 ml) was added to a solution of Diels-Alder adduct **2a** (3.5 g, 18 mmol) in EtOH (200 ml) at room temperature and this was followed by portionwise addition of zinc powder (26.6 g, 407 mmol). The mixture was stirred at room temperature for 12 hours and then filtered. The filtrate was treated with ^i^Pr_2_NEt (36.4 g, 282 mmol) and di-*tert*-butyldicarbonate (7.0 g, 32mmol). The mixture was stirred at 25 ^o^C for 20 hours. The solvent was removed at reduced pressure to about 10 ml. The reduced solution was partitioned between ethyl acetate (50 ml), sat. NaHCO_3_ (30 ml) and water (30 ml). The organic layer was separated, dried (MgSO_4_) and removal of the solvent under reduced pressure gave a white solid which was purified by flash chromatography using petroleum ether/Et_2_O (7:3) as the eluent to give the title compound **2b** (406 mg, 89%) as a white solid, mp 88-90^o^C; ν_max_ (KBr disk): 3353, 2982, 1737,1700 cm^-1^; δ_H_ (500 MHz, CDCl_3_): 1.27 (3H, *t*, J = 7.2 Hz, OCH_2_CH_3_), 1.30 (9H, *s*, OC(CH_3_)_3_), 2.05 (1H, *d*, J = 3.0 Hz, H-2), 4.20 (2H, *q*, J = 7.2 Hz, OCH_2_CH_3_), 4.32 (1H, br, NH), 4.52 (1H, br, H-3), 5.08 (1H, br, H-4), 5.11 (1H, *s*, H-1), 6.46 (1H, *d*, J = 5.7 Hz, H-5), 6.59 (1H, *d*, J = 5.7 Hz, H-6); δ_C_ (125 MHz, CDCl_3_): 14.5 (OCH_2_CH_3_), 28.7 (OC(CH_3_)_3_), 52.6 (C-2), 53.6 (C-3), 61.6 (OCH_2_CH_3_), 75.0 (C-4), 79.3 (OC(CH_3_)_3_), 82.4 (C-1), 134.7 (C-5), 138.0 (C-6), 157.4 (NCO_2_), 172 ( CO_2_C_2_H_5_); *m/z* (EI): 283 (M^+^), 57 (100%).

**Ethyl *exo*-3-*tert*-butoxycarbonylamino-7-oxabicyclo[2.2.1]hept-5-ene-*endo*-2-carboxylate 3b**

In an identical fashion *exo* nitro Diels-Alder adduct **3a** could be converted to the corresponding carbamate **3b**; Found C, 59.41; H, 7.46; N, 4.94; C_14_H_21_NO_5_ requires C, 95.35; H, 7.47; N, 4.94; mp 50-52 ^o^C, ν_max_ (KBr disk): 3291, 2978, 1738, 1703 cm^-1^; δ_H_ (500 MHz, CDCl_3_): 1.24 (3H, *t*, J = 7.2 Hz, OCH_2_CH_3_), 1.45 (9H, *s*, OC(CH_3_)_3_), 2.69 (1H, *t*, J = 4.8 and 3.2 Hz, H-2), 4.02 (1H, *br*, H-3), 4.12 (2H, *q*, J = 7.2 Hz, OCH_2_CH_3_), 4.78 (1H, *s*, H-4), 4.82 (1H, *br*, NH), 5.12 (1H, *d*, J = 4.8 Hz, H-1), 6.37 (1H, *dd*, J = 5.2 and 1.6 Hz, H-5), 6.46 (1H, *dd*, J = 5.2 and 1.6 Hz, H-6); δ_C_ (125 MHz, CDCl_3_): 14.0 (OCH_2_CH_3_), 28.2 (OC(CH_3_)_3_), 52.1 (C-2), 54.3 (C-3), 60.8 (OCH_2_CH_3_), 78.4 (C-1), 79.6 (C-3), 85.1 (C-4), 135.2 (C-6), 135.4 (C-5), 155.3 (NCO_2_), 170.2 (COC_2_H_5_); MS (EI): *m/z* 283(M^+^), 57(100%).

**Ethyl *trans*-6-tert-butoxycarbonylamino-5-hydroxy-1,3-cyclohexadiene-1-carboxylate 4**:

To a solution of KHMDS (530 mg, 2.64 mmol) in THF (10 ml) at –50 ^o^C was added a solution of adduct **2b** (250 mg, 0.88 mmol) in THF (2.5 ml). The solution was then warmed up to room temperature (20 minutes) and quenched with a mixture of ethyl acetate and ethanol (50 ml, 19:1). The mixture was washed with sat. NH_4_Cl (25 ml), dried (MgSO_4_) and concentrated under reduced pressure. The resulting residue was purified by flash chromatography eluting with petroleum ether/ethyl acetate mixture (3:2) to give cyclohexadiene **4** (359 mg, 72%) as a pale yellow gum. Found: C, 59.20; H, 7.53; N, 4.80%; Calc. for C_14_H_21_NO_5_ C, 59.35; H, 7.47; N, 4.94%; ν_max_ (KBr disk): 2979, 2931, 1716, 1700 1584 cm^-1^; δ_H_ (500 MHz, CDCl_3_): 1.28 (3H, *t*, J = 7.0 Hz, OCH_2_CH_3_), 1.42 (9H, *s*, OC(CH_3_)_3_), 4.20 (2H, *q*, J = 7.0 Hz, OCH_2_CH_3_), 4.36 (1H, *s*, H-5), 4.48 (1H, br, NH), 4.76(1H, *m*, H-6), 6.26 (2H, *m*, H-3 and 4), 7.17 (1H, *d*, J = 4.8 Hz, H-2); δ_C_ (125 MHz, CDCl_3_): 14.1 (OCH_2_CH_3_), 28.3 (OC(CH_3_)_3_), 50.0 (C-6), 60.8 (OCH_2_CH_3_), 67.7(C-5), 80.0 (OC(CH_3_)_3_), 124.5 (C-3), 132.6 (C-2), 133.5 (C-4), 155.4 (NCO_2_), 165.9 (CO_2_C_2_H_5_); MS m/z (CI): 284 (M^+^ + 1);.

**Ethyl *syn*-6-*tert*-butoxycarbonylamino-5-hydroxy-1,3-cyclohexadiene-1-carboxylate 5**: To a solution of KHMDS (1060 mg, 5.28 mmol) in THF (10 ml) at –50 ^o^C was added a solution of adduct **3b** (500 mg, 1.67 mmol) in THF (2.5 ml). The solution was then warmed up to room temperature (20 minutes) and quenched with a mixture of ethyl acetate and ethanol (50 ml, 19:1). The mixture was washed with sat. NH_4_Cl (25 ml), dried (MgSO_4_) and concentrated under reduced pressure. The resulting residue was purified by flash chromatography eluting with petroleum ether/ethyl acetate mixture (3:2) to give cyclohexadiene **5** (340 mg, 68%) as a pale yellow gum; ν_max_ (KBr disk): 3389 (br), 2982, 1689, 1523 cm^-1^; δ_H_ (500 MHz, CDCl_3_): 1.28 (3H, *t*, J = 7.0 Hz, OCH_2_CH_3_), 1.42 (9H, *s*, OC(CH_3_)_3_), 3.48 (1H, br, OH), 4.22 (2H, *q*, J = 7.0 Hz, OCH_2_CH_3_), 4.64 (1H, *t*, J = 7.5 Hz, H-5), 4.67 (1H, br, NH), 4.77 (1H, *d*, J = 7.5 Hz, H-6), 6.05 (1H, *t*, J = 5.5 Hz, H-3), 6.11 (1H, *d*, J = 7.5 Hz, H-4), 7.10 (1H, *d*, J = 5.5 Hz, H-2); δ_C_ (125 MHz, CDCl_3_): 14.4 (OCH_2_CH_3_), 28.5 (OC(CH_3_)_3_), 46.9 (C-6), 61.6 (OCH_2_CH_3_), 71.4 (C-5), 81.6 (OC(CH_3_)_3_), 122.2 (C-3), 127.9 (C-1), 135.2 (C-2), 138.8 (C-4), 157.5 (NCO_2_), 166.1 (CO_2_); *m/z* (CI): 284 (MH^+^, 13%), 182 (100%); HRMS (CI): Found MH^+^, 284.1486; C_14_H_22_NO_5_^+^ requires *M,* 284.1498.

**Ethyl *anti*-*anti*-3-acetoxy-2-*tert*-butoxycarbonylaminocyclohexanecarboxylate 6**:

Following the same protocol as described for **7**, acylation and reduction of cyclohexene **4** afforded ACHC derivative **6** as a colourless oil (75%) following purification by column chromatography; ν_max_ (liq. film): 3367 (br), 2938, 1727, 1688, 1535 cm^-1^; δ_H_ (500MHz, CDCl_3_): 1.23 (3H, *t*, J = 7.5 Hz, OCH_2_CH_3_), 1.40 (9H, *s*, OC(CH_3_)_3_), 1.52 and 1.77 (2H, *m*, HH-5), 1.54 and 1.87 (2H, *m*, HH-6), 1.55 and 2.05 (2H, *m*, HH-4), 2.08 (3H, *s*, CH_3_CO), 2.36 (1H, *dt*, J = 12.3 and 3.9 Hz, H-1), 3.22 (1H, br, OH), 3.42 (1H, br, H-3), 3.49 (1H, br, H-2), 4.12 (2H, *q*, J = 7.5 Hz, OCH_2_CH_3_), 4.81 (1H, br, NH); δ_C_ (125 MHz, CDCl_3_): 14.4 (OCH_2_CH_3_), 23.1 (C-6), 28.5 (OC(CH_3_)_3_), 28.8 (C-5), 33.9 (C-4), 48.6 (C-1), 58.3 (C-2), 60.9 (OCH_2_CH_3_), 74.1 (C-3), 80.2 (OC(CH_3_)_3_), 157.0 (NCO_2_), 173.4 (CO_2_Et); *m/z* (CI): 330 (MH^+^, 53%), 232 (100%).

**Ethyl *syn*-*syn*-3-acetoxy-2-*tert*-butoxycarbonylaminocyclohexanecarboxylate 7**:

To a solution of cyclohexadiene **5** (500 mg, 1.77 mmol) in CH_2_Cl_2_ anhydrous pyridine (0.5 ml, 6.45 mmol) was added acetic anhydride (0.5 ml, 5.30 mmol). The reaction mixture was then stirred at room temperature for 20 hours. The solvent was removed under reduced pressure and the crude product was purified by flash chromatography eluting with petroleum ether/ethyl acetate (4:1) to give *ethyl syn-5-acetoxy-6-tert-butoxycarbonylamino-1,3-cyclohexadiene-1-carboxylate* as a yellow solid (449 mg, 78%); m.p. 76-78^o^C; ν_max_ (KBr disk): 3330, 2984, 1710,1522 cm^-1^; δ_H_ (500 MHz, CDCl_3_): 1.27 (3H, *t*, J = 7.0 Hz, OCH_2_CH_3_), 1.41 (9H, *s*, OC(CH_3_)_3_), 2.09 (3H, *s*, CH_3_CO), 4.25 (2H, *q*, J = 7.0 Hz, OCH_2_CH_3_), 4.56 (1H, *d*, J = 10.0 Hz, NH), 5.00 (1H, *dd*, J = 10.0 and 7.5 Hz, H-6), 5.66 (1H, *d*, J = 7.5 Hz, H-5), 6.00 (1H, *d*, J = 9.5 Hz, H-4), 6.16 (1H, *dd*, J = 9.5 and 5.5 Hz, H-3), 7.12 (1H, *d*, J = 5.5 Hz, H-2), δ_C_ (125 MHz, CDCl_3_): 14.3 (OCH_2_CH_3_), 21.2 (CH_3_CO), 28.5 (OC(CH_3_)_3_), 43.7 (C-6), 61.2 (OCH_2_CH_3_), 71.6 (C-5), 79.7 (OC(CH_3_)_3_), 124.0 (C-3), 128.6 (C-1), 134.0 (C-2), 134.3 (C-4), 155.2 (NCO_2_), 165.8 (CO_2_), 170.5 (CH_3_CO); *m/z* (CI): 326 (MH^+^, 100%).

To a solution of *ethyl syn-5-acetoxy-6-tert-butoxycarbonylamino-1,3-cyclohexadiene-1-carboxylate* (250 mg, 0.77 mmol) in ethanol (20 ml) was added 10% Pd/C (100 mg) and the solution was stirred under hydrogen for 48 h. The reaction mixture was then filtered and the filtrate was concentrated under reduced pressure to give ACHC derivative **7** as a white solid (243 mg, 96%); m.p. 53-55 ^o^C; ν_max_ (KBr disk): 3373, 2982, 1744, 1715, 1524 cm^-1^; δ_H_ (500 MHz, CDCl_3_): 1.23 (3H, *t*, J = 7.0 Hz, OCH_2_CH_3_), 1.40 (9H, *s*, OC(CH_3_)_3_), 1.56 (2H, *m* HH-5), 1.72 (2H, *m*, HH-4), 1.81 (2H, *m*, HH-6), 1.99 (3H, *s*, CH_3_CO), 2.62 (1H, *dd*, J = 10.5 and 5.5 Hz, H-1), 4.16 (2H, *q*, J = 7.0 Hz, OCH_2_CH_3_), 4.47 (1H, *m*, H-2), 4.83 (1H, *m*, H-3), 4.98 (1H, *br*, NH); δ_C_ (125 MHz, CDCl_3_): 14.3 (OCH_2_CH_3_), 21.2 (C-6), 21.3 (CH_3_CO), 22.9 (C-4), 26.5 (C-5), 28.5 (OC(CH_3_)_3_), 43.9 (C-1), 49.8 (C-2), 60.9 (OCH_2_CH_3_), 72.4 (C-3), 79.6 (OC(CH_3_)_3_), 155.6 (NCO_2_), 170.5 and 172.7 (carbonyls); *m/z* (ES^+^): 352 (MNa^+^, 100%).

**N-Benzyl-[*anti*-*anti*-3-acetoxy-2-*tert*-butoxycarbonylaminocyclohexane-1-carboxyl]amide 8**:

A solution of *anti-anti-3-acetoxy-2-tert-butoxycarbonylaminocyclohexanecarboxylic acid* (250 mg, 1.09 mmol), benzylamine (0.14 ml, 1.31 mmol), and triethylamine in CH_2_Cl_2_ (10 ml) was treated with HATU (501 mg, 1.31 mmol) and the reaction was stirred for 16 h. 1 M HCl (20 ml) was added to the reaction mixture and the layers were separated. The organic layer was then dried (MgSO_4_), concentrated under reduced pressure and recrystalisation from ethyl acetate afforded **8** as a white solid (360 mg, 87%); m.p. 182-184 ^o^C; ν_max_ (KBr disk): 3450, 2933, 1734, 1689,1644 cm^-1^; δ_H_ (500 MHz, CD_3_OD): 1.39 (9H, *s*, OC(CH_3_)_3_), 1.45 (2H, *m*, HH-4), 1.57 (2H, *m*, HH-5), 1.83 (2H, *m*, HH-6), 2.00 (3H, *s*, CH_3_CO), 2.45 (1H, *dt*, J = 11.6 and 3.2 Hz, H-1), 3.74 (1H, *t*, J = 11.6 Hz, H-2), 4.25 (1H, *d*, J = 14.8 Hz, NCH_2_Ph), 4.47 (1H, *d*, J = 14.8 Hz, NCH_2_Ph), 4.75 (1H, *m*, H-3), 7.28 (5H, *m*, aromatic protons); δ_C_ (100 MHz, CD_3_OD): 19.9 (CH_3_CO), 22.8 (C-5), 27.6 (OC(CH_3_)_3_), 29.1 (C-6), 30.8 (C-4), 42.9 (NCH_2_Ph), 49.4 (C-1), 54.6 (C-2), 74.9 (C-3), 78.7 (OC(CH_3_)_3_), 126.9, 127.3, 128.4, 138.7 (Ph carbons), 156.5 (NCO_2_), 174.1 (NCO), 174.4 (CH_3_CO); *m/z* (ES_+_): 413 (MNa^+^)

**N-Cyclohexyl-[*anti*-*anti*-3-acetoxy-2-*tert*-butoxycarbonylamino-cyclohexanecarboxyl]amide 9**

Employing the same procedure as that described for the synthesis of **8** reaction of *anti-anti-3-acetoxy-2-tert-butoxycarbonylamino-cyclohexanecarboxylic acid* (250 mg, 1.09 mmol) with cyclohexylamine afforded **9** as a white solid (292 mg, 72%); m.p. 260-262 ^o^C; ν_max_ (KBr disk): 3311, 2933, 1738, 1693, 1645, 1547 cm^-1^; δ_H_ (400 MHz, DMSO): 1.12 (1H, *m*, H-4’), 1.23 (4H, *m*, HH-3’ and 5’), 1.35 (9H, *s*, OC(CH_3_)_3_), 1.41 (2H, *m*, HH-5), 1.63 (4H, *m*, HH-2’ and 6’), 1.84 (4H, *m*, HH-4 and 6), 2.01 (3H, *s*, CH_3_CO), 2.53 (1H, *dd*, J = 9.0 and 10.2 Hz, H-1), 3.48 (2H, *m*, H-1’ and H-2), 4.57 (1H, *m*, H-3); δ_C_ (100 MHz, DMSO): 21.4 (C-3’), 23.2 (C-6), 23.6 (C-4’), 24.8 (C-5’), 25.9 (C-5), 28.8 (OC(CH_3_)_3_), 31.2 (C-2’), 31.5 (C-6’), 32.8 (C-4), 38.9 (C-1), 46.4 (C-2), 48.3 (C-1’), 76.0 (C-3), 79.3 (OC(CH_3_)_3_), 152.3 (NCO_2_), 166.3 (NCO), 170.5 (CH_3_CO); *m/z* (ES^+^): 405 (MNa^+^).

**6-*tert*-butoxycarbonylamino-3,4-epoxy-5-hydroxycyclohex-1-ene-1-carboxylate**

To a solution of diene **4** (1.00 g, 3.53 mmol) in CH_2_Cl_2_ (20 ml) was added mCPBA (1.23 g, 7.06 mmol) and NaHCO_3_ (890 mg, 10.6 mmol). The mixture was stirred at room temperature for 36 hr. The mixture was then partitioned between concentrated NaHCO_3_ solution (25 ml) and ethyl acetate (25 ml) and the layers were separated. The aqueous layer was extracted with ethyl acetate (2 x 25 ml) and the organic extractions were mixed, dried (MgSO_4_) and concentrated under reduced pressure to give the crude product of 9:1 mixture of epoxides **10** as a colourless oil (721 mg, 68%) and **11** as a white gum (90 mg, 9%). The two isomers were separated by flash chromatography eluting with petroleum ether/ethyl acetate (7:4). *Ethyl anti-anti-6-tert-butoxycarbonylamino-3,4-epoxy-5-hydroxycyclohex-1-ene-1-carboxylate* **10**: ν_max_ (thin film): 3439 (br), 2979, 1718, 1499 cm^-1^; δ_H_ (500 MHz, CDCl_3_): 1.27 (3H, *t*, J = 7.0 Hz, OCH_2_CH_3_), 1.40 (9H, *s*, OC(CH_3_)_3_), 3.54 (1H, *t*, J = 4.0 Hz, H-3), 3.74 (1H, br, H-4), 4.24 (2H, *q*, J = 7.0 Hz, OCH_2_CH_3_), 4.41 (1H, br, H-5), 4.73 (1H, *d*, J = 10.0 Hz, NH), 4.87 (1H, *d*, J = 10.0 Hz, H-6), 7.32 (1H, *d*, J = 4.0 Hz, H-2); δ_C_ (125 MHz, CDCl_3_): 14.3 (OCH_2_CH_3_), 28.6 (OC(CH_3_)_3_), 46.4 (C-3), 49.3 (C-6), 58.1 (C-4), 61.4 (OCH_2_CH_3_), 67.0 (C-5), 80.2 (OC(CH_3_)_3_), 133.0 (C-1), 137.3 (C-2), 155.3 (NCO_2_), 165 (CO_2_); *m/z* (CI): 300 (MH^+^, 25%), 261 (100%). *Ethyl syn-anti-6-tert-butoxycarbonylamino-3,4-epoxy-5-hydroxycyclohex-1-ene-1-carboxylate* **11**: ν_max_ (thin film): 3435 (br), 2972, 1725, 1492 cm^-1^; δ_H_ (500 MHz, CDCl_3_): 1.28 (3H, *t*, J = 7.0 Hz, OCH_2_CH_3_), 1.41 (9H, *s*, OC(CH_3_)_3_), 3.50 (1H, *t*, J = 4.0 Hz, H-3), 3.70 (1H, *dd*, J = 4.0 and 1.5 Hz, H-4), 4.18 (4H, *m*, OCH_2_CH_3_, H-5, and H-6), 5.49 (1H, br, NH), 6.92 (1H, *d*, J = 4.0 Hz, H-2); δ_C_ (125 MHz, CDCl_3_): 14.3 (OCH_2_CH_3_), 28.5 (OC(CH_3_)_3_), 48.3 (C-3), 52.5 (C-6), 55.6 (C-4), 61.5 (OCH_2_CH_3_), 71.6 (C-5), 80.6 (OC(CH_3_)_3_), 130.1 (C-1), 133.4 (C-2), 156.7 (NCO_2_), 165.5 (CO_2_); *m/z* (CI): 300 (MH^+^, 20%), 166 (100%).

**Ethyl *anti*-*anti*-5-acetoxy-6-*tert*-butoxycarbonylamino-3,4-epoxy-5-acetoxycyclohex-1-ene-1-carboxylate 12**:

To a solution of cyclohexadiene **3** (500 mg, 1.77 mmol) in anhydrous pyridine (5 ml, 64.5 mmol) was added acetic anhydride (5 ml, 53.0 mmol). The reaction mixture was then stirred at room temperature for 20 hours. 2M HCl (20 ml) and ethyl acetate (20 ml) were added and the resulting layers were separated. The aqueous layer was extracted with ethyl acetate (2 x 20 ml) and the organic layers were combined, dried (MgSO_4_) and concentrated under reduced pressure. The crude product was purified by flash chromatography eluting with petroleum ether/ethyl acetate (4:1) to give *ethyl anti-5-acetoxy-6-tert-butoxycarbonylamino-1,3-cyclohexadiene-1-carboxylate* as a white solid (550 mg, 96%), mp 42-44^o^C; Found: C, 58.79; H, 7.11; N, 4.30; Calc. for C_16_H_23_NO_6_; C, 59.06; H, 7.13; N, 4.31%; ν_max_ (KBr disk): 3285, 2983, 1737, 1717, 1679, 1648, 1527 cm^-1^; δ_H_ (500 MHz, CDCl_3_): 1.30 (3H, t, J = 7.0 Hz, OCH_2_CH_3_), 1.42 (9H, s, OC(CH_3_)_3_), 2.00 (3H, s, CH_3_CO), 4.28 (2H, q, J = 7.0 Hz, OCH_2_CH_3_), 4.37 (1H, br, NH), 4.46 (1H, br, H-6), 5.25 (1H, br, H-5), 6.34 (2H, br, H-3 and 4), 7.17(IH, br, H-2); δ_C_ (125 MHz, CDCl_3_): 14.1 (OCH_2_CH_3_), 20.8 (CH_3_CO), 28.2 (OC(CH_3_)_3_), 46.2 (C-6), 61.0 (OCH_2_CH_3_), 68.8 (C-5), 80.0 (OC(CH_3_)_3_), 126.6 and 127.8 (C-3 and 4) 128.7 (C-2), 133.1 (C-1), 154.6 (NCO_2_), 165.5 (CH_3_CO), 169.8 (CO_2_C_2_H_5_); MS m/z (CI): 326 (MH^+^, 100%). To a solution of *ethyl anti-5-acetoxy-6-tert-butoxycarbonylamino-1,3-cyclohexadiene-1-carboxylate* (200 mg, 0.59 mmol) in CH_2_Cl_2_ (10 ml) was added mCPBA (310 mg, 1.77 mmol) and NaHCO_3_ (300 mg, 3.52 mmol). The mixture was stirred at room temperature for 36 hours. The mixture was then partitioned between concentrated NaHCO_3_ solution (25 ml) and ethyl acetate and the layers were separated. The aqueous layer was extracted with ethyl acetate (2 x 25 ml) and the organic extractions were combined, dried (MgSO_4_) and concentrated under reduced pressure to give the crude product. Flash chromatography eluting with petroleum ether/ethyl acetate (7:3) gave the title epoxide **12** as a colourless oil (136 mg, 65%); Found: C, 56.27; H, 6.85; N, 4.11%; Calc. for C_16_H_23_NO_7_ C, 56.30; H, 6.79; N, 4.10%; ν_max_ (thin film): 3442, 2979, 1653, 1494 cm^-1^; δ_H_ (400 MHz, CDCl_3_): 1.25 (3H, *t*, J = 7.2 Hz, OCH_2_CH_3_), 1.38 (9H, *s*, OC(CH_3_)_3_), 2.00 (3H, *s*, CH_3_CO), 3.50 (1H, *dd*, J = 7.5 and 3.9 Hz, H-3), 3.79 (1H, *br*, H-4), 4.19 (2H, *q*, J = 7.2 Hz, OCH_2_CH_3_), 4.16 (1H, *d*, J = 9.9 Hz, NH), 4.87 (1H, *d*, J = 9.9 Hz, H-6), 5.32 (1H, *br*, H-5), 7.28 (1H, *br*, H-2); δ_C_ (100 MHz, CDCl_3_): 14.3 (OCH_2_CH_3_), 21.0 (CH_3_CO), 28.5 (OC(CH_3_)_3_), 46.3 (C-3), 46.8 (C-6), 55.6 (C-4), 61.5 (OCH_2_CH_3_), 68.7 (C-5), 80.0 (OC(CH_3_)_3_), 133.4 (C-1), 136.6 (C-2), 154.4 (NCO_2_), 164.8 (CO_2_C_2_H_5_), 169.9 (CH_3_CO); MS *m/z* (CI): 342 (MH^+^), 303 (100%).

**Ethyl *syn*-*syn*-6-*tert*-butoxycarbonylamino-3,4-epoxy-5-hydroxycyclohex-1-ene-1-carboxylate 13**

To a solution of diene **5** (1.00 g, 3.53 mmol) in CH_2_Cl_2_ (20 ml) was added *m*CPBA (1.23 g, 7.06 mmol) and NaHCO_3_ (890 mg, 10.6 mmol). The mixture was stirred at room temperature for 36 hr. The mixture was then partitioned between concentrated NaHCO_3_ solution (25 ml) and ethyl acetate (25 ml) and the layers were separated. The aqueous layer was extracted with ethyl acetate (2 x 25 ml) and the organic extractions were mixed, dried (MgSO_4_) and concentrated under reduced pressure to give the crude product **13** as a white gum (865 mg, 82%); ν_max_ (KBr disk): 3437, 2979, 1720, 1498 cm^-1^; δ_H_ (500 MHz, CDCl_3_): 1.27 (3H, *t*, J = 7.5 Hz, OCH_2_CH_3_), 1.41 (9H, *s*, OC(CH_3_)_3_), 3.24 (1H, *br*, OH), 3.53 (1H, *dd*, J = 4.5 and 4.0 Hz, H-3), 3.67 (1H, *m*, H-4), 4.21 (3H, *m*, OCH_2_CH_3_ and H-5), 4.80 (1H, *d*, J = 8.5 Hz, NH), 4.98 (1H, *dd*, J = 8.5 and 4.5 Hz, H-6), 7.16 (1H, *d*, J = 4.0 Hz, H-2); δ_C_ (125 MHz, CDCl_3_): 14.3 (OCH_2_CH_3_), 28.5 (OC(CH_3_)_3_), 47.8 (C-6), 48.3 (C-2), 59.0 (C-4), 61.6 (OCH_2_CH_3_), 68.4 (C-5), 80.2 (OC(CH_3_)_3_), 135.6 (C-1), 136.8 (C-2), 156.9 (NCO_2_), 164.7 (CO_2_); *m/z* (ES^+^): 322 (MNa^+^, 100%).

**Ethyl *anti*-*anti*-*anti*-3,4-diacetoxy-2-*tert*-butylcarbonylaminocyclohexane-1-carboxylate** **14**:

To a solution of epoxide **12** (100 mg, 0.29 mmol) in ethanol (10 ml) was added 10% Pd/C (20 mg) and the suspension was stirred under hydrogen for 48 h. The suspension was then filtered and the filtrate was concentrated under reduced pressure. The residue was dissolved in pyridine (2.5 ml) and treated with Ac_2_O (2.5 ml). After stirring for 24 h, 1 M HCl (10 ml) was added and the layers were separated. The organic layer was dried (MgSO_4_), concentrated under pressure and subjected to column chromatography eluting with petroleum ether/ethyl acetate (7:3) to give **14** as a colourless gum (98 mg, 99%); ν_max_ (KBr disk): 3319, 2979, 1732, 1692, 1543 cm^-1^; δ_H_ (500 MHz, CDCl_3_): 1.23 (3H, *t*, J = 7.0 Hz, OCH_2_CH_3_), 1.37 (9H, *s*, OC(CH_3_)_3_), 1.70 (1H, *m*, H-6), 1.94 (1H, *m*, H-6), 2.00 (3H, *s*, CH_3_CO), 2.02 (3H, *s*, CH_3_CO), 2.14 (2H, *m*, HH-5), 2.47 (1H, *dt*, J = 9.0 and 3.0 Hz, H-1), 3.88 (1H, *m*, H-2), 4.13 (2H, *q*, J = 7.0 Hz, OCH_2_CH_3_), 4.88 (1H, *d*, J = 9.5 Hz, H-3), 4.98 (1H, *m*, H-4); δ_C_ (125 MHz, CDCl_3_): 14.3 (OCH_2_CH_3_), 20.9 and 21.2 (2 x CH_3_CO), 24.9 (C-6), 28.4 (OC(CH_3_)_3_), 28.8 (C-5), 48.5 (C-1), 54.1 (C-2), 61.3 (OCH_2_CH_3_), 72.6 (C-3), 75.0 (C-4), 79.9 (OC(CH_3_)_3_), 155.0 (NCO_2_), 170.3, 171.0, 172.2 (CO_2_ and 2 x CH_3_CO); *m/z* (CI): 405 (MNH_4_^+^, 71%), 388 (MH^+^, 36%), 349 (100%).

**Ethyl *anti*-*anti*-*syn*-3,4-diacetoxy-2-*tert*-butylcarbonylaminocyclohexane-1-carboxylate** **15**:

To a solution of epoxide **11** (100 mg, 0.33 mmol) in ethanol (10 ml) was added Pd/C (20 mg) under a hydrogen atmosphere and the mixture was stirred for 48 h. The solvent was then removed under reduced pressure and the residue was dissolved in pyridine (2.5 ml) and treated with Ac_2_O (2.5 ml). After stirring for 24 h, 2 M HCl (10 ml) was added to the reaction mixture and the layers were separated. The organic layer was dried (MgSO_4_), concentrated under reduced pressure and subjected to column chromatography eluting with petroleum ether/ethyl acetate (7:3) to give **15** as a white gum (107 mg, 84%); ν_max_ (KBr disk): 3385, 2975, 1737, 1525 cm^-1^; δ_H_ (500 MHz, CDCl_3_): 1.25 (3H, *t*, J = 7.2 Hz, OCH_2_CH_3_), 1.39 (9H, *s*, OC(CH_3_)_3_), 1.64 (1H, *m*, H-5), 1.81 (1H, *m*, H-6), 1.95 (2H, *m*, H-5 and 6), 2.01 (3H, *s*, CH_3_CO), 2.13 (3H, *s*, CH_3_CO), 2.51 (1H, *t*, J = 11.5 Hz, H-1), 4.16 (3H, *m*, OCH_2_CH_3_ and H-2), 4.45 (1H, *d*, J = 9.5 Hz, NH), 4.86 (1H, *dd*, J = 11.5 and 2.5 Hz, H-3), 5.32 (1H, br, H-4); δ_C_ (125 MHz, CDCl_3_) 14.4 (OCH_2_CH_3_), 21.0 (CH_3_CO), 21.5 (CH_3_CO), 23.1 (C-6), 27.8 (C-5), 28.5 (OC(CH_3_)_3_), 48.8 (C-1), 50.7 (C-2), 61.2 (OCH_2_CH_3_), 69.4 (C-4), 73.3 (C-3), 79.7 (OC(CH_3_)_3_), 155.1 (NCO_2_), 170.6, 171.O, 172.8 (carbonyls); *m/z* (CI): 388 (MH^+^, 100%).

**Ethyl *syn*-*syn*-*syn*-3-acetoxy-2-*tert*-butylcarbonylamino-4-hydroxycyclohexane-1-carboxylate** **16**:

Following acylation by the standard protocol, epoxide **13** (100 mg, 0.29 mmol) in ethanol (10 ml) was added 10% Pd/C (20 mg) and the suspension was stirred under hydrogen for 48 h. The suspension was then filtered and the filtrate was concentrated under reduced pressure to give **16** as a colourless gum (97 mg, 97%) ν_max_ (KBr disk): 3438, 3374, 2975, 1729, 1699, 1513 cm^-1^; δ_H_ (500 MHz, CDCl_3_): 1.23 (3H, *t*, J = 7.0 Hz, OCH_2_CH_3_), 1.40 (9H, *s*, OC(CH_3_)_3_), 1.57 (1H, *m*, H-5), 1.63 (1H, *m*, H-6), 2.02 (2H, *m*, H-5 and 6), 2.07 (3H, *s*, CH_3_CO), 2.60 (1H, *m*, J = 11.5 and 3.5 Hz, H-1), 4.07 (2H, *m*, H-4 and OH), 4.13 (2H, *q*, J = 7.0 Hz, OCH_2_CH_3_), 4.52 (1H, br, H-2), 4.85 (1H, *m*, H-3), 5.72 (1H, br, NH); δ_C_ (125 MHz, CDCl_3_): 14.3 (OCH_2_CH_3_), 17.4 (C-6), 21.2 (CH_3_CO), 28.5 (OC(CH_3_)_3_), 28.9 (C-5), 44.4 (C-1), 50.0 (C-2), 61.0 (OCH_2_CH_3_), 68.9 (C-4), 72.6 (C-3), 79.4 (OC(CH_3_)_3_), 155.9 (NCO_2_), 170.2 (CO_2_), 172.3 (CH_3_CO); *m/z* (ES^+^): 368 (MNa^+^).

**Ethyl *endo*-3-*tert*-butoxycarbonylamino-5,6-O,O-bis(tert-butyldimethylsilyl)-5,6-dihydroxy-7-oxabicyclo[2,2,1]heptane-*exo*-2-carboxylate 17**

The diol (500 mg, 1.58 mmol), obtained following standard dihydroxylation of **2b**, was dissolved in anhydrous DMF (20 ml) and treated with DMAP (583 mg, 4.78 mmol) and TBSCl (600 mg, 4.00 mmol). The mixture was then stirred over night, quenched with sat. NH_4_Cl solution (10 ml), and extracted with ethyl acetate (10 ml x 3). The combined organic extracts were washed with water (10 ml), dried (MgSO_4_) and concentrated to leave a residue, which was purified by flash chromatography eluting with petroleum ether/(CH_3_CH_2_)_2_O (7:3) to give disilyl ether **17** as a white solid (860 mg, 86%), mp 150-152^o^C; Found: C, 57.14; H, 9.38; N, 2.59% Calc. for C_26_H_51_NO_7_Si_2_: C, 57.21; H, 9.42; N, 2.57%; ν_max_ (KBr disk): 3372, 2959, 1737, 1699, 1677, 1520 cm^-1^; δ_H_ (500 MHz, CDCl_3_): 0.09, 0.12 (12H, *s*, 2 x Si(CH_3_])_2_), 0.91 (18H, *s*, 2 x SiC(CH_3_)_3_), 1.26 (3H, *t*, J = 7.0 Hz, OCH_2_CH_3_), 1.44 (9H, *s*, OC(CH_3_)_3_), 2.08 (1H, *d*, J = 4.6 Hz, H-2), 3.92 (1H, *d*, J = 5.3 Hz, H-6), 4.09 (1H, br, H-3), 4.18 (1H, *q*, J = 7.0 Hz, OCH_2_CH_3_), 4.25 (1H, br, H-5), 3.47 (1H, *s*, H-1), 4.52 (1H, br, H-4), 4.63 (1H, br, NH); δ_C_ (125 MHz, CDCl_3_): -4.9, -5.0 (2 x Si(CH_3_)_2_), 14.1 (OCH_2_CH_3_), 18.5, 18.6 (2 x SiC(CH_3_)_3_), 26.0 (SiC(CH_3_)_3_), 28.3 (OC(CH_3_)_3_), 49.8 (C-2), 53.2 (C-3), 61.4 (OCH_2_CH_3_), 71.6 (C-5), 77.3 (C-6), 80.3 (C-4), 80.4 (OC(CH_3_)_3_), 85.4 (C-1), 155.4 (NCO_2_), 171.1 (CO_2_C_2_H_5_); MS (EI): *m/z* 547(M^+^), 376 (100 %).

**Ethyl *syn*-*anti*-*anti*-6-*tert*-butoxycarbonylamino-3,4,5-trihydroxycyclohex-1-ene-1-carboxylate 18**

Using the general procedure for OsO_4_ dihydroxylation, Me_3_NO.H_2_O (93 mg, 0.84 mmol), 4% wt. OsO_4_ in water (50 ml) and diene **4** (120 mg, 0.42 mmol) gave a crude product which was subjected to column chromatography eluting with petroleum ether/ethyl acetate (7:3) to give **18** as a colourless oil (104 mg, 78%); ν_max_ (thin film): 3424, 2980, 1710,1513 cm^-1^; δ_H_ (400MHz, CDCl_3_): 1.26 (3H, *t*, J = 6.8 Hz, OCH_2_CH_3_), 1.40 (9H, *s*, OC(CH_3_)_3_), 3.83 (1H, br, H-4), 4.05 (1H, br, H-5), 4.18 (2H, *q*, J = 6.8 Hz, OCH_2_CH_3_), 4.35 (1H, *m*, H-6), 4.45 (1H, br, H-3), 5.61 (1H, br, NH), 6.82 (1H, *s*, H-2); δ_C_ (100 MHz, CDCl_3_): 14.3 (OCH_2_CH_3_), 28.6 (OC(CH_3_)_3_), 51.5 (C-6), 61.3 (OCH_2_CH_3_), 66.0 (C-3), 70.5 (C-4), 71.7 (C-5), 80.2 (OC(CH_3_)_3_), 131.7 (C-1), 132.2 (C-2), 156.3 (NCO_2_), 166.3 (CO_2_Et); *m/z* (CI): 318 (MH^+^, 49%), 182 (100%).

**Ethyl *syn*-*syn*-*anti*-6-amino-3,4-*bis*-(*tert*-butyldimethylsilyloxy)-5-hydroxycyclohex-1-ene-1-carboxylate 19**

To a solution of LiHMDS (231 mg, 1.38 mmol) in THF (20 ml) at –50^o^C was added a solution of oxanorbornene **17** (250 mg, 0.46 mmol) in THF (5 ml). The solution was stirred at 25^o^C for 2 hours. The reaction mixture was then quenched with ethyl acetate/ethanol (9:1) and stirred for 5 minutes. Saturated NH_4_Cl solution was added to the reaction mixture and the resulting two layers were separated. The organic layer was dried (MgSO_4_) and concentrated under reduced pressure to give the crude product. Flash chromatography eluting with ethyl acetate/petroleum ether (3:2) afforded **19** as a light yellow oil (98 mg, 48%); ν_max_ (KBr disk): 3410, 2985, 1734, 1538 cm^-1^; δ_H_ (400 MHz, CDCl_3_): 0.14 (12H, *s*, 2 x Si(CH_3_)_3_), 0.90 (9H, *s*, SiC(CH_3_)_3_), 0.91 (9H, *s*, SiC(CH_3_)_3_), 1.33 (3H, *t*, J = 7.2Hz, OCH_2_CH_3_), 3.25 (1H, br, OH), 4.02 (1H, *dd*, J = 5.6 and 5.0 Hz, H-5), 4.08 (1H, *dd*, J = 7.4 and 5.6 Hz, H-6), 4.13 (1H, *t*, J = 2.4 Hz, H-4), 4.26 (2H, *q*, J = 7.2 Hz, OCH_2_CH_3_), 4.41 (1H, *dd*, J = 3.2 and 2.4 Hz, H-3), 4.68 (2H, br, NH_2_), 6.87 (1H, d, J = 3.2 Hz, H-2); δ_C_ (100 MHz, CDCl_3_): 0.1 (Si(CH_3_)_2_), 18.8 (OCH_2_CH_3_), 23.0 (SiC(CH_3_)_3_), 30.6 (SiC(CH_3_)_3_), 55.8 (C-6), 66.3 (OCH_2_CH_3_), 73.8 (C-3), 76.4 (C-4), 77.4 (C-5), 118.7 (C-1), 132.1 (C-2), 170.2 (CO_2_). *m/z* (CI): 446 (MH+, 8%), 297 (100%).

**Ethyl *syn*-*syn*-*syn*-3,4,5-triacetoxy-6-*tert*-butoxycarbonylaminocyclohex-1-ene-1-carboxylate 20**:

To a solution of **5** (120 mg, 0.23 mmol) in acetone (15 ml) was added Me_3_NO.H_2_O (52 mg, 0.47 mmol) and 4 wt % OsO_4_ in water (50 ml). The resulting mixture was stirred and monitored by TLC until all the starting material was consumed (12 hours). The solvent was then removed under reduced pressure and the residue was dissolved in ethyl acetate (10 ml). The acetate solution was washed with saturated NaHSO_3_ (5 ml) and the aqueous phase was extracted with ethyl acetate (5 ml x 3). The organic layers were combined, dried over MgSO_4_ and the solvent was removed under reduced pressure. Subsequently, the residue was dissolved in pyridine (2.5 ml, 31.0 mmol) and acetic anhydride (2.5 ml, 26.5 mmol) and stirred at room temperature for 16 h. Water (20 ml) was then added and the mixture was stirred for further 3 h. The mixture was extracted with ethyl acetate (3 x 20 ml). The combined organic extracts were washed with 2M HCl (30 ml), sat. NaHCO_3_ (30 ml) and water (30 ml), dried (MgSO_4_) and concentrated under reduced pressure. The residue was purified by flash chromatography eluting with petroleum ether/ethyl acetate (7:3) to afford title ester **20** (110 mg, 75%) as a white gum; ν_max_ (KBr disk): 3463, 2980, 1755, 1721, 1507 cm^-1^; δ_H_ (500 MHz, CDCl_3_): 1.28 (3H, *t*, J = 7.0 Hz, OCH_2_CH_3_), 1.44 (9H, *s*, OC(CH_3_)_3_), 2.04 (3H, *s*, CH_3_CO), 2.06 (3H, *s*, CH_3_CO), 2,14 (3H, *s*, CH_3_CO), 4.26 (2H, *q*, J = 7.0 Hz, OCH_2_CH_3_), 4.85 (1H, br, NH), 5.07 (2H, *m*, H-4 and 6), 5.61 (2H, *m*, H-3 and 5), 6.75 (1H, br, C-2); δ_C_ (125 MHz, CDCl_3_): 14.3 (OCH_2_CH_3_), 20.8 (2 x CH_3_CO), 21.1 (CH_3_CO), 28.5 (OC(CH_3_)_3_), 45.1 (C-6), 61.7 (OCH_2_CH_3_), 66.8 (C-4), 67.9 (C-3), 68.7 (C-5), 79.7 (OC(CH_3_)_3_), 132.2 (C-1), 136.1 (C-2), 155.2 (NCO_2_), 164.5 (CO_2_), 169.6, 169.7, 169.9 (3 x CH_3_CO); *m/z* (ES^+^): 466 (MNa^+^); HRMS (ES^+^) Found MNa^+^, 466.1679. C_20_H_29_NO_10_Na^+^ requires *M,* 466.1689.

**Ethyl *anti*-*anti*-*anti*-*syn*-3,4,5-triacetoxy-2-*tert*-butoxycarbonylaminocyclohexanecarboxylate 21**:

**166** (100mg, 0.30 mmol) was dissolved in pyridine (2.5 ml, 31.0 mmol) and acetic anhydride (2.5 ml, 26.5 mmol) and stirred at room temperature for 16 h. Water (20 ml) was then added and the mixture was stirred for further 3 h. The mixture was extracted with ethyl acetate (3 x 20 ml). The combined organic extracts were washed with 2M HCl (30 ml), sat. NaHCO_3_ (30 ml) and water (30 ml), dried (MgSO_4_) and concentrated under reduced pressure. The residue was purified by flash chromatography eluting with petroleum ether/ethyl acetate (7:2) to give *ethyl syn-anti-anti-3,4,5-triacetoxy-6-tert-butoxycarbonylamino-cyclohex-1-ene-1-carboxylate* as a thick yellow oil (127 mg, 68%). Found: C, 54.23; H, 6.52; N, 3.18; Calc. for C_20_H_29_NO_10_: C, 54.42; H, 6.59; N, 3.16. δ_H_ (500 MHz, CDCl_3_): 1.28 (3H, *t*, J = 7.2 Hz, OCH_2_CH_3_), 1.42 (9H, *s*, OC(CH_3_)_3_), 2.07 (9H, *s*, 3 x CH_3_CO), 4.23 (2H, *q*, J = 7.2 Hz, OCH_2_CH_3_), 4.42 (1H, br, NH), 4.70 (1H, br, H-6), 5.26 (1H, br, H-5), 5.32 (1H, *dd*, J = 6.6 and 4.2 Hz, H-4), 5.67 (1H, *t*, J = 3.6 Hz , H-3), 6.76 (1H, br, H-2); δ_C_ (125 MHz, CDCl_3_): 14.0 (OCH_2_CH_3_), 20.5 and 20.7 (3 x CH_3_CO), 28.2 (OC(CH_3_)_3_), 48.3 (C-6), 61.4 (OCH_2_CH_3_), 65.6 (C-3), 66.1 (C-4), 70.2 (C-5), 79.9 (OC(CH_3_)_3_), 133.0 (C-1), 134.3 (C-2), 154.4 (NCO_2_), 164.6 (CO_2_C_2_H_5_), 169.1, 169.3 and 169.8 (3 x CH_3_CO); *m/z* (CI): 444 (MH^+^). Using the general hydrogenation procedure, *ethyl syn-anti-anti-3,4,5-triacetoxy-6-tert-butoxycarbonylamino-cyclohex-1-ene-1-carboxylate* was converted to the triacetate **21** δ_H_ (500 MHz, CDCl_3_): 1.29 (3H, *t*, J = 7.0 Hz, OCH_2_CH_3_), 1.40 (9H, *s*, OC(CH_3_)_3_), 1.74 and 1.96 (2H, *m*, 6-HH), 2.93 (1H, *dd*, J = 8.5 and 4.4 Hz, H-1), 3.94 (1H, br, H-5), 4.11 (2H, *q*, J = 7.0 Hz, OCH_2_CH_3_), 4.22 (1H, br, H-4), 4.53 (1H, br, H-3), 4.59 (1H, br, H-2), 5.64 (1H, br, NH); δ_C_ (125 MHz, CDCl_3_): 14.4 (OCH_2_CH_3_), 21.0 (3 x CH_3_CO), 24.9 (C-6), 28.4 (OC(CH_3_)_3_), 41.8 (C-1), 48.5 (C-5), 54.1 (C-2), 61.5 (OCH_2_CH_3_), 77.0 (C-4), 79.8 (H-3), 81.2 (OC(CH_3_)_3_), 155.0 (NCO_2_), 169.8 (CO_2_), 171.1, 172.8 and 173.2 (3 x CH_3_CO); *m/z* (CI): 463 (MNH_4_^+^, 3%), 446 (MH^+^, 5%), 346 (100%), 232 (93%). HRMS (ES+): Found MNa^+^ 468.1849, C_17_H_25_NO_9_Na requires *M,* 468.1846.

**Ethyl *anti*-*anti*-*syn*-*syn*-3,4,5-triacetoxy-2-N-acetylaminocyclohexane-1-carboxylate 22**

*Tetra*-n-butylammonium fluoride (0.5 ml of 1M solution in THF, 0.5 mmol) was added to a stirred solution of disilyl ether **19** (90 mg, 0.20 mmol) in THF (2.5 ml). After 16 hours, the mixture was concentrated under reduced pressure to afford yellow oil. The oil was dissolved in pyridine (1.5 ml, 18.6 mmol) and acetic anhydride (1.5 ml, 15.9 mmol) and the solution was stirred for 20 hours. Water (5 ml) and ethyl acetate (10 ml) were added to the reaction mixture and the resulting layers were separated. The aqueous layer was extracted with ethyl acetate (2 x 10 ml). The organic extracts were combined, washed with 2M HCl (10 ml), saturated NaHCO_3_ (10 ml), water (10ml), dried (MgSO_4_) and concentrated under reduced pressure. The residue was purified by flash chromatography eluting with petroleum ether/ethyl acetate (7:3) to give *ethyl syn-syn-anti-3,4,5-triacetoxy-6-N-acetylaminocyclohex-1-ene-1-carboxylate* as a yellow gum (41 mg, 52%). ν_max_ (KBr disk): 3343, 2977, 1711, 1639, 1521 cm^-1^; δ_H_ (500 MHz, CDCl_3_): 1.27 (3H, *t*, J = 7.2 Hz, OCH_2_CH_3_), 1.97 (3H, *s*, CH_3_CO), 2.03 (3H, *s*, CH_3_CO), 2.06 (3H, *s*, CH_3_CO), 2.15 (3H, *s*, CH_3_CO), 4.25 (2H, *q*, J = 7.2 Hz, OCH_2_CH_3_), 5.13 (1H, *dd*, J = 5.5 and 2.0 Hz, H-5), 5.44 (1H, br H-6), 5.62 (1H, br, H-4), 5.65 (2H, *m*, H-3 and NH), 6.79 (1H, br, H-2); δ_C_ (125 MHz, CDCl_3_): 14.3 (OCH_2_CH_3_), 20.7 (CH_3_CO), 20.8 (CH_3_CO), 21.2 (CH_3_CO), 23.5 (CH_3_CO), 43.6 (C-6), 61.8 (OCH_2_CH_3_), 66.4 (C-5), 67.8 (C-3), 68.8 (C-4), 132.0 (C-1), 136 (C-2), 164.3 , 169.2, 169.6, 169.8 (carbonyls); *m/z* (CI): 386 (MH^+^, 100%). To a solution of *ethyl syn-syn-anti-3,4,5-triacetoxy-6-N-acetylaminocyclohex-1-ene-1-carboxylate* (100 mg, 0.26 mmol) in ethanol (10 ml) was added 10% Pd/C (20 mg) and the suspension was stirred under hydrogen for 24 h. The suspension was then filtered and the filtrate was concentrated under reduced pressure to give **22** as a colourless gum (98 mg, 99%); ν_max_ (KBr disk): 3408, 2982, 1741, 1668, 1538 cm^-1^; δ_H_ (400 MHz, CDCl_3_): 1.23 (3H, *s*, J = 7.2 Hz, OCH_2_CH_3_), 1.97 (3H, *s*, CH_3_CO), 2.00 (3H, *s*, CH_3_CO), 2.01 (3H, *s*, CH_3_CO), 2.10 (2H, *m*, HH-6), 2.19 (3H, *s*, CH_3_CO), 2.75 (1H, *dd*, J = 10.2 and 9.2 Hz, H-1), 4.12 (2H, *q*, J = 7.2 Hz, OCH_2_CH_3_), 4.91 (1H, *m*, H-3), 4.92 (1H, *m*, H-2), 4.96 (1H, *m*, H-4), 5.54 (1H, *m*, H-5), 6.24 (1H, br, NH); δ_C_ (100 MHz, CDCl_3_): 14.2 (OCH_2_CH_3_), 20.9 (CH_3_CO), 21.0 (CH_3_CO), 21.2 (2 x CH_3_CO), 23.7 (C-6), 38.5 (C-1), 47.2 (C-2), 61.7 (OCH_2_CH_3_), 68.0 (C-4), 68.6 (C-3), 70.6 (C-5), 169.1, 169.6, 169.8, 170.0, 170.9 (carbonyls); *m/z* (CI): 388 (MH^+^, 100%). HRMS (ES^+^): Found: 410.1388; C_17_H_25_NO_9_Na requires *M*, 410.1427.

**Ethyl *syn*-*syn*-*syn*-*syn*-3,4,5-triacetoxy-2-*tert*-butoxycarbonylaminocyclohexane carboxylate 23**:

To a solution of cyclohexene **20** (100 mg, 0.26 mmol) in ethanol (10 ml) was added 10% Pd/C (20 mg) and the suspension was stirred under hydrogen for 24 h. The suspension was then filtered and the filtrate was concentrated under reduced pressure to give **23** as a yellow oil (98 mg, 98%); ν_max_ (thin film): 3455, 2979, 1747, 1513 cm^-1^; δ_H_ (500 MHz, CDCl_3_): 1.22 (3H, *t*, J = 7.5 Hz, OCH_2_CH_3_), 1.41 (9H, *s*, OC(CH_3_)_3_), 2.00 (3H, *s*, CH_3_CO), 2.01 (6H, *s*, 2 x CH_3_CO), 2.06 (2H, *m*, HH-6), 2.16 (3H, *s*, CH_3_CO), 2.73 (1H, *m*, H-1), 4.22 (2H, *q*, J = 7.5 Hz, OCH_2_CH_3_), 4.63 (1H, *m*, H-2), 4.87 (2H, *m*, H-4 and 5), 5.16 (1H, *d*, J = 11.0 Hz, NH), 5.53 (1H, *s*, H-3); δ_C_ (125 MHz, CDCl_3_): 14.2 (OCH_2_CH_3_), 20.8 (CH_3_CO), 21.0 (2 x CH_3_CO), 22.8 (C-6), 28.5 (OC(CH_3_)_3_), 41.8 (C-1), 48.7 (C-2), 61.5 (OCH_2_CH_3_), 68.6 (C-4), 68.7 (C-5), 70.5 (C-3), 79.7 (OC(CH_3_)_3_), 155.8 (NCO_2_), 169.4, 169.7, 170.1, 170.8 (carbonyls); *m/z* (ES^+^): 468 (MNa^+^); HRMS (ES^+^): Found MNa^+^, 468.1860. C_20_H_31_NO_10_Na^+^ requires *M,* 468.1846.

**Ethyl *anti*-*anti*-*anti*-*anti*-3,4,5-triacetoxy-2-N-acetylaminocyclohexane-1-carboxylate 24**

A solution of epoxide **12** (270 mg, 0.79 mmol) in H_2_O/Acetone (1:1) was treated with HClO_4_ (5 ml, 0.08 mmol). The solution was stirred at room temperature for 24 h and the solvent was removed under reduced pressure. The residue was stirred in pyridine (5 ml) and acetic anhydride (5 ml) for 24 h and then partitioned between 2M HCl (20 ml) and ethyl acetate (20 ml). The organic layer was washed with saturated NaHCO_3_ (20 ml), dried (MgSO_4_) and concentrated under reduced pressure. The resulting residue was columned using petroleum ether/ ethyl acetate (7:3) as the eluting solvent to give *ethyl anti-anti-anti-3,4,5-triacetoxy-2-N-acetylaminocyclohex-1-ene-1-carboxylate* **27** as a pale yellow gum (226 mg, 74%); ν_max_ (KBr disk): 3387, 2935, 1752, 1663, 1537 cm^-3^; δ_H_ (500 MHz, CDCl_3_): 1.27 (3H, *t*, J = 7.0 Hz, OCH_2_CH_3_), 1.93 (3H, *s*, CH_3_CO), 2.05 (3H, *s*, CH_3_CO), 2.06 (3H, *s*, CH_3_CO), 2.08 (3H, *s*, CH_3_CO), 4.21 (2H, *q*, J = 7.0 Hz, OCH_2_CH_3_), 5.01 (1H, *m*, H-6), 5.32 (2H, *m*, H-4 and H-5), 5.58 (1H, br, NH), 6.72 (1H, *d*, J = 4.5 Hz, H-2); δ_C_ (125 MHz, CDCl_3_): 14.3 (OCH_2_CH_3_), 20.9, 21.0 and 23.4 (4 x CH_3_CO), 49.7 (C-6), 61.7 (OCH_2_CH_3_), 69.7 (C-3), 70.4 and 71.3 (C-4 and C-5), 132.0 (C-1), 135.0 (C-2), 164.6 (CO_2_), 169.6, 169.7, 170.1 and 170.4 (4 x CH_3_CO); *m/z* (CI): 386 (MH^+^), 208 (100%). To a solution of *ethyl anti-anti-anti-3,4,5-triacetoxy-2-N-acetylaminocyclohex-1-ene-1-carboxylate* (100 mg, 0.26 mmol) in ethanol (10 ml) was added 10% Pd/C (20 mg) and the suspension was stirred under hydrogen for 24 h. The suspension was then filtered and the filtrate was concentrated under reduced pressure to give **24** as a white gum (98 mg, 98%); ν_max_ (KBr disk): 3247, 2947, 1741, 1660, 1565 cm^-1^; δ_H_ (500 MHz, CDCl_3_): 1.23 (3H, *t*, J = 7.0 Hz, OCH_2_CH_3_), 1.88 (1H, *m*, H-6), 1.90 (3H, *s*, CH_3_CON), 2.01 (3H, *s*, CH_3_CO), 2,03 (3H, *s*, 2 x CH_3_CO), 2.28 (1H, *t*, J = 13.0 Hz, H-6), 2.57 (1H, *dt*, J = 13.0 and 3.5 Hz, H-1), 4.12 (2H, *q*, J = 7.0 Hz, OCH_2_CH_3_), 4.33 (1H, *q*, J = 10.0 Hz, H-2), 4.88 (1H, *m*, H-5), 4.96 (1H, *t*, J = 10.0 Hz, H-3), 5.21 (1H, *t*, J = 10.0 Hz, H-4), 5.49 (1H, *d*, J = 10.0 Hz, NH); δ_C_ (125 MHz, CDCl_3_): 14.3 (OCH_2_CH_3_), 20.9 and 21.1 (3 x CH_3_CO), 23.4 (CH_3_CON), 30.3 (C-6), 44.4 (C-1), 52.5 (C-2), 61.8 (OCH_2_CH_3_), 70.6 (C-5), 72.9 (C-4), 73.2 (C-3), 169.8, 170.4, 170.8 and 171.3 (carbonyls); *m/z* (CI): 388 (MH^+^, 100%); HRMS (ES^+^): Found: 410.1467; C_17_H_25_NO_9_Na requires M^+^, 410.1427.

**Ethyl *anti*-*anti*-*syn*-*anti*-3,4,5-triacetoxy-2-N-acetylaminocyclohexane-1-carboxylate 25**

A solution of epoxide **11** (270 mg, 0.79 mmol) in H_2_O/Acetone (1:1) was treated with HClO_4_ (5 ml, 0.08 mmol). The solution was stirred at room temperature for 24 h and the solvent was removed under reduced pressure. The residue was stirred in pyridine (5 ml) and acetic anhydride (5 ml) for 24 h and then partitioned between 2M HCl (20 ml) and ethyl acetate (20 ml). The organic layer was washed with saturated NaHCO_3_ (20 ml), dried (MgSO_4_) and concentrated under reduced pressure. The resulting residue was columned using petroleum ether/ ethyl acetate (7:3) as the eluting solvent to give *ethyl anti-syn-anti-3,4,5-triacetoxy-6-N-acetylaminocyclohex-1-ene-1-carboxylate* as a pale yellow gum (226 mg, 74%); ν_max_ (KBr disk): 3379, 2996, 1744, 1663, 1543 cm^-1^; δ_H_ (500 MHz, CDCl_3_): 1.26 (3H, *t*, J = 7.0 Hz, OCH_2_CH_3_), 1.97 (3H, *s*, CH_3_CO), 2.01 (3H, *s*, CH_3_CO), 2.06 (3H, *s*, CH_3_CO), 2.08 (3H, *s*, CH_3_CO), 4.27 (2H, *q*, J = 7.0 Hz, OCH_2_CH_3_), 4.97 (1H, *dd*, J = 8.5 and 3.5 Hz, H-6), 5.28 (1H, *dd*, J = 8.5 and 2.5 Hz, H-4), 5.39 (1H, *dd*, J = 8.5 and 3.5 Hz, H-5), 5.57 (1H, *dd*, J = 8.5 and 2.5 Hz, H-3), 5.85 (1H, *d*, J = 8.5 Hz, NH), 6.87 (1H, *d*, J = 2.5 Hz, H-2); δ_C_ (125 MHz, CDCl_3_): 14.3 (OCH_2_CH_3_), 21.0 (CH_3_CO), 21.1 (2 x CH_3_CO), 23.3 (CH_3_CO), 47.7 (C-6), 61.8 (OCH_2_CH_3_), 68.9 (C-4), 69.0 (C-3), 70.4 (C-5), 130.5 (C-1), 138.0 (C-2), 164.6, 169.6, 169.7 and 170.5 (carbonyls); *m/z* (CI): 386 (MH^+^, 100%).To a solution of *ethyl anti-syn-anti-3,4,5-triacetoxy-6-N-acetylaminocyclohex-1-ene-1-carboxylate* (100 mg, 0.26 mmol) in ethanol (10 ml) was added 10% Pd/C (20 mg) and the suspension was stirred under hydrogen for 48 h. The suspension was then filtered and the filtrate was concentrated under reduced pressure to give **195** as a colourless gum (98 mg, 99%); ν_max_ (KBr disk): 3379, 2981, 1743, 1549 cm^-1^; δ_H_ (500 MHz, CDCl_3_):1.24 (3H, t, J = 7.0 Hz, OCH_2_CH_3_), 1.90 (3H, *s*, CH_3_CO), 2.00 (3H, *s*, CH_3_CO), 2.03 (1H, *m*, H-6), 2.10 (3H, *s*, CH_3_CO), 2.16 (3H, *s*, CH_3_CO), 2.17 (1H, *m*, H-6), 2.71 (1H, *dt*, J = 11.5 and 4.0 Hz, H-1), 4.14 (2H, *q*, J = 7.0 Hz, OCH_2_CH_3_), 4.52 (1H, *q*, J = 11.5 Hz, H-2), 5.04 (1H, *m*, H-5), 5.14 (1H, *dd*, J = 11.5 and 3.0 Hz, H-3), 5.25 (1H, *t*, J = 3.0 Hz, H-4), 5.50 (1H, *d*, J = 11.5 Hz, NH); δ_C_ (125 MHz, CDCl_3_): 14.4 (OCH_2_CH_3_), 21.0 (CH_3_CO), 21.3 (CH_3_CO), 23.5 (CH_3_CO), 27.8 (C-6), 43.8 (C-1), 49.4 (C-2), 61.6 (OCH_2_CH_3_), 68.6 (C-5), 68.8 (C-4), 70.3 (C-3), 169.4, 169.8, 170.0, 171.3 and 171.8 (carbonyls); *m/z* (CI): 388 (MH^+^, 100%); HRMS (ES^+^): Found M^+^, 410.1417. C_17_H_25_NO_9_ requires *M*, 410.1427.

**Ethyl *anti*-*syn*-*syn*-*anti*-3,4,5-triacetoxy-2-N-acetylaminocyclohexane-1-carboxylate 26**

A solution of epoxide **13** (270 mg, 0.79 mmol) in H_2_O/Acetone (1:1) was treated with HClO_4_ (5 ml, 0.08 mmol). The solution was stirred at room temperature for 24 h and the solvent was removed under reduced pressure. The residue was stirred in pyridine (5 ml) and acetic anhydride (5 ml) for 24 h and then partitioned between 2M HCl (20 ml) and ethyl acetate (20 ml). The organic layer was washed with saturated NaHCO_3_ (20 ml), dried (MgSO_4_) and concentrated under reduced pressure. The resulting residue was columned using petroleum ether/ ethyl acetate (7:3) as the eluting solvent to give *ethyl anti-syn-syn-3,4,5-triacetoxy-2-N-acetylaminocyclohex-1-ene-1-carboxylate* as a pale yellow gum (218 mg, 72%); ν_max_ (KBr disk):3377, 2989, 1745, 1675, 1535 cm^-1^; δ_H_ (500 MHz, CDCl_3_): 1.26 (3H, *t*, J = 7.0 Hz, OCH_2_CH_3_), 1.96 (3H, *s*, CH_3_CON), 2.08 (3H, *s*, CH_3_CO), 2.09 (3H, *s*, CH_3_CO), 2.10 (3H, *s*, CH_3_CO), 4.23 (2H, *q*, J = 7.0 Hz, OCH_2_CH_3_), 5.22 (1H, *dd*, J = 5.5 and 2.0 Hz, H-4), 5.46 (2H, *m*, H-5 and 6), 5.58 (2H, *m*, H-3 and NH), 6.82 (1H, *d*, J = 2.5 Hz, H-2); δ_C_ (125 MHz, CDCl_3_): 14.3 (OCH_2_CH_3_), 21.0 (CH_3_CO) 21.1 (2 x CH_3_CO), 23.4 (CH_3_CON), 44.8 (C-6), 61.8 (OCH_2_CH_3_), 68.0 (C-3), 68.4 (C-5), 70.6 (C-4), 133.2 (C-1), 135.0 (C-2), 164.9 (CH_3_CON), 169.1, 169.3, 169.9, 170.0 (carbonyls); *m/z* (ES^+^): 408 (MNa^+^, 100%). To a solution of *ethyl anti-syn-syn-3,4,5-triacetoxy-2-N-acetylaminocyclohex-1-ene-1-carboxylate* (100 mg, 0.26 mmol) in ethanol (10 ml) was added 10% Pd/C (20 mg) and the suspension was stirred under hydrogen for 24 h. The suspension was then filtered and the filtrate was concentrated under reduced pressure to give **26** as a white solid (98 mg, 98 %); m.p. 196-198^o^C; ν_max_ (KBr disk): 3271, 2986, 1747, 1651, 1556 cm^-1^; δ_H_ (500 MHz, CDCl_3_): 1.23 (3H, *t*, J = 7.0 Hz, OCH_2_CH_3_), 1.79 (2H, *m*, H-6), 1.91 (3H, *s*, CH_3_CO), 1.97 (3H, *s*, CH_3_CON), 2.03 (3H, *s*, CH_3_CO), 2.16 (3H, *s*, CH_3_CO), 2.77 (1H, *m*, H-1), 4.12 (2H, *q*, J = 7.0 Hz, OCH_2_CH_3_), 4.50 (1H, *m*, H-2), 5.05 (2H, *m*, H-4 and 5), 5.50 (1H, br, H-3), 5.56 (1H, *d*, J = 8.0 Hz, NH); δ_C_ (125 MHz, CDCl_3_): 14.3 (OCH_2_CH_3_), 20.8 (CH_3_CO), 21.2 (CH_3_CO), 21.2 (CH_3_CO), 23.3 (CH_3_CON), 30.0 (C-6), 42.7 (C-1), 49.6 (C-2), 61.7 (OCH_2_CH_3_), 68.5 (C-5), 71.5 (C-3), 71.8 (C-4), 169.3, 169.9, 170.0, 170.7, 171.4 (carbonyls); *m/z* (ES^+^): 410 (MNa^+^).

**Ethyl *anti*-*anti*-*anti*-5-acetoxy-6-*tert*-butoxycarbonylamino-3-chloro-4-hydroxy-1-cyclohexene-1-carboxylate 28**:

To solution of epoxide **12** (240 mg, 0.75mmol) in dichloromethane was added acetic acid (120 ml, 2.10 mmol) and ZnCl_2_ (102mg, 0.75mmol). The mixture was stirred at room temperature until all the starting material was consumed (TLC ca. 12 h). The solvent was remove under reduced pressure and the residue was subjected to column chromatography eluting with petroleum ether/ethyl acetate (7:3) to give **28** as a white solid (200 mg, 83%); m.p 158-160^o^C; ν_max_ (KBr disk): 3447 (br), 2979, 1765, 1703, 1652, 1536 cm^-1^; δ_H_ (500 MHz, CDCl_3_): 1.30 (3H, *t*, J = 7.0 Hz, OCH_2_CH_3_), 1.42 (9H, *s*, OC(CH_3_)_3_), 2.10 (3H, *s*, CH_3_CO), 2.86 (1H, br, OH), 4.12 (1H, *m*, H-4), 4.24 (2H, *q*, J = 7.0 Hz, OCH_2_CH_3_), 4.61 (2H, *m*, H-3 and 6), 4.80 (1H, br, NH), 5.16 (1H, br, H-5), 6.81 (1H, *s*, H-2); δ_C_ (125 MHz, CDCl_3_): 14.3 (OCH_2_CH_3_), 21.1 (CH_3_CO), 28.5 (OC(CH_3_)_3_), 50.3 (C-3), 57.3 (C-6), 61.7 (OCH_2_CH_3_), 72.8 (C-4), 73.7 (C-5), 80.9 (OC(CH_3_)_3_), 131.7 (C-1), 136.5 (C-2), 155.2 (NCO_2_), 165.0 (CO_2_), 170.9 (CH_3_CO); MS *m/z* (ES^+^): 400 (MNa^+^).

**Ethyl 2-hydroxy-5-oxo-4,9-dioxa-6-azatricyclo[3,3,1,1^1,8^]decane-10-carboxylate 29**

A solution of epoxide **11** (200 mg, 1.01 mmol) in AcOH/H_2_O (9:1, 10 ml) was stirred at room temperature for 30 minutes. The reaction was quenched with sat. NaHCO_3_ (10 ml). The resulting solution was extracted with ethyl acetate (10 ml x 3). The combined organic layers were dried (MgSO_4_) and concentrated under reduced pressure to afford the crude product. Purification of the crude product by flash chromatography eluting with ethyl acetate/hexane (7:3) gave **29** (120 mg, 75%) as a white solid, mp 37-39^o^C; Found: C, 49.22; H, 5.15; N, 5.84%; Calc. for C_10_H_13_NO_6_ C, 49.38; H, 5.39; N, 5.76%; ν_max_ (KBr disk): 3448 (br), 2979, 1733, 1681, 1463 cm^-1^; δ_H_ (400 MHz, CDCl_3_): 1.29 (3H, t, J = 7.2 Hz, OCH_2_CH_3_), 2.59 (1H, d, J = 2.4 Hz, H-10), 3.83 (1H, d, J = 1.2 Hz, H-2), 4.21 (2H, q, J = 7.2 Hz, OCH_2_CH_3_), 4.32 (1H, m, H-7), 4.54 (1H, t, J = 1.2 and 1.5 Hz, H-8), 4.61 (2H, m, H-1 and 3), 5.98 (1H, br, NH); δ_C_ (100 MHz, CDCl_3_): 14.1 (OCH_2_CH_3_), 53.1 (C-7), 54.3 (C-10), 61.9 (OCH_2_CH_3_), 67.2 (C-8), 75.6 (C-2), 79.1 (C-2), 85.1 and 85.5 (C-1 and 3), 150.1 (C-5), 170.1 (CO_2_C_2_H_5_); MS (EI): *m/z* 243 (M^+^), 98 (100%).

**X-ray crystallography**

X-ray diffraction experiments were carried out on a Bruker 3-circle diffractometers with CCD area detectors SMART 1K (for **26** and **28**) or APEX (for **2b**), using graphite-monochromated Mo-*K*α radiation ($\bar{\lambda}$=0.71073 Å) from sealed tube (**26** and **28**) or 60W Mo-target microfocus Bede Microsource® X-ray generator with glass polycapillary X-ray optics (**2b**). Low temperature of the crystals was maintained with Cryostream (Oxford Cryosystems) open-flow N_2_ gas cryostats. The structures were solved by direct methods and refined by full-matrix least squares (non-hydrogen atoms in anisotropic, all H atoms in isotropic approximation) against *F*^2^ of all reflections, using SHELXL 6.12 programs (Bruker AXS, Madison, Wisconsin, USA, 2001). Crystal data and experimental details are listed in Table 1. In **26** the O(5) atom is disordered between two positions with occupancies 85% and 15% [O(5’)].

Fig.1. Molecular structure of **2b** (50% thermal ellipsoids)

Fig. 2. Molecular structure of **26**

Fig. 3. Molecular structure of **28**

**Table 1.** Crystal Data

| Compound | **2b** | **26** | **28** |
| --- | --- | --- | --- |
| CCDC number | 604000 | 604001 | 604002 |
| Formula | C_14_H_21_NO_5_ | C_17_H_25_NO_9_ | C_16_H_24_ClNO_7_ |
| FW | 283.32 | 387.38 | 377.81 |
| Temperature, K | 120 | 120 | 120 |
| Crystal system | monoclinic | orthorhombic | monoclinic |
| Space group | P2_1_/*c* (#14) | P*bca* (#61) | P2_1_/*c* (#14) |
| *a*, Å | 8.5458(6) | 19.335(3) | 9.902(1) |
| *b*, Å | 11.243(1) | 9.4556(16) | 8.788(1) |
| *c*, Å | 15.371(1) | 21.594(4) | 22.520(3) |
| β, ˚ | 96.10(1) | 90 | 96.14(1) |
| *U*, Å^3^ | 1468.5(2) | 1749.6(3) | 1948.4(4) |
| *Z* | 4 | 8 | 4 |
| *D*_calc_ (g/cm^3^) | 1.281 | 1.304 | 1.288 |
| μ, mm^-1^ | 0.10 | 0.11 | 0.23 |
| Reflections measured | 14745 | 41426 | 17291 |
| Unique reflections | 3366, 2994* | 4563, 3273* | 5173, 3731* |
| *R*_int_ | 0.025 | 0.064 | 0.041 |
| *R*_1_ | 0.040* | 0.046* | 0.037* |
| w*R*_2_ | 0.107 | 0.114 | 0.090 |

*R*_1_=Σ||*F*_c_|−|*F*_o_||/Σ|*F*_o_|, *wR*_2_=[Σ*w*(*F*_o_^2^− *F*_c_^2^)2/Σ*w*(*F*_o_^2^)^2^]^1/2^

* For reflections with *F*^2^≥2σ(*F*^2^)
